# Opportunities and Challenges of Li_2_C_4_O_4_ as Pre‐Lithiation Additive for the Positive Electrode in NMC622||Silicon/Graphite Lithium Ion Cells

**DOI:** 10.1002/advs.202201742

**Published:** 2022-07-07

**Authors:** Aurora Gomez‐Martin, Maike Michelle Gnutzmann, Egy Adhitama, Lars Frankenstein, Bastian Heidrich, Martin Winter, Tobias Placke

**Affiliations:** ^1^ MEET Battery Research Center, Institute of Physical Chemistry University of Münster Corrensstr. 46 Münster 48149 Germany; ^2^ International Graduate School for Battery Chemistry, Characterization, Analysis, Recycling and Application (BACCARA) University of Münster Corrensstr. 40 Münster 48149 Germany; ^3^ Helmholtz‐Institute Münster, IEK‐12 Forschungszentrum Jülich GmbH Corrensstr. 46 Münster 48149 Germany

**Keywords:** active lithium losses, cathode additives, lithium ion batteries, pre‐lithiation, silicon

## Abstract

Silicon (Si)‐based negative electrodes have attracted much attention to increase the energy density of lithium ion batteries (LIBs) but suffer from severe volume changes, leading to continuous re‐formation of the solid electrolyte interphase and consumption of active lithium. The pre‐lithiation approach with the help of positive electrode additives has emerged as a highly appealing strategy to decrease the loss of active lithium in Si‐based LIB full‐cells and enable their practical implementation. Here, the use of lithium squarate (Li_2_C_4_O_4_) as low‐cost and air‐stable pre‐lithiation additive for a LiNi_0.6_Mn_0.2_Co_0.2_O_2_ (NMC622)‐based positive electrode is investigated. The effect of additive oxidation on the electrode morphology and cell electrochemical properties is systematically evaluated. An increase in cycle life of NMC622||Si/graphite full‐cells is reported, which grows linearly with the initial amount of Li_2_C_4_O_4_, due to the extra Li^+^ ions provided by the additive in the first charge. Post mortem investigations of the cathode electrolyte interphase also reveal significant compositional changes and an increased occurrence of carbonates and oxidized carbon species. This study not only demonstrates the advantages of this pre‐lithiation approach but also features potential limitations for its practical application arising from the emerging porosity and gas development during decomposition of the pre‐lithiation additive.

## Graphical abstract

Pre‐lithiation is a promising approach to compensate for active lithium losses in lithium ion battery cells. Herein, the beneficial impact of Li_2_C_4_O_4_ as pre‐lithiation additive on prolonging the cycle life of NMC622||Si/graphite full‐cells is reported. This study also points out possible detrimental effects of decomposition products such as releasing gases and remaining cathode porosity on cell performance.

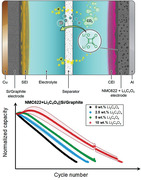

## 1 Introduction

Lithium ion batteries (LIBs) represent the state‐of‐the‐art (SOTA) power sources for portable electronic devices and are the most promising candidate for electro mobility and stationary energy storage applications.^[^ ^1^ ^]^ However, the growing energy demand for LIBs for electric vehicle applications requires improvements in terms of increased energy density (≥750 Wh L^−1^ at cell level) and reduced costs along the whole battery value chain.^[^ ^1^, ^2^ ^]^ Silicon (Si) stands out as the next‐generation high‐capacity negative electrode (= anode) material replacing SOTA graphite anodes for LIBs due to its high specific capacity of 3579 mAh g^−1^, low average de‐lithiation potential (≈0.4 V vs Li|Li^+^), low voltage hysteresis, and low cost of precursor materials.^[^ ^3^ ^]^ Unfortunately, Si suffers from several challenges that hinder its broad application in commercial LIB cells so far:^[^ ^4^ ^]^ significant volume changes upon (de‐)lithiation leading to pulverization of Si particles, detachment of composite electrode constituents from the current collector, continuous breakage and (re‐)formation of the solid electrolyte interphase (SEI) resulting in ongoing active lithium losses (ALL) from the positive electrode ( = cathode), electrolyte consumption and therefore a decrease in cell energy density and lifetime.^[^ ^5^ ^]^

Apart from advanced design of the morphology of Si particles and electrode formulation as well as the use of optimized electrolytes,^[^ ^6^ ^]^ pre‐lithiation has emerged as a highly appealing method to minimize ALL by introducing additional lithium into the cell prior to operation.^[^ ^7^ ^]^ Several pre‐lithiation techniques have been proposed so far, each with its benefits and challenges for the practical use.^[^ ^7a^ ^]^ With respect to direct pre‐lithiation of the anode, chemical,^[^ ^8^ ^]^ electrochemical,^[^ ^9^ ^]^ and contact pre‐lithiation^[^ ^10^ ^]^ have been extensively explored. Alternatively, indirect pre‐lithiation of the anode can also be achieved in situ during the first charge with the help of lithium excess provided by using over‐lithiated cathode active materials or by the incorporation of additives in the cathode during electrode processing.^[^ ^7a,b^ ^]^ Over‐lithiation of the cathode material can be conducted electrochemically in a lithium metal cell^[^ ^11^ ^]^ or chemically using reactive compounds such as n‐butyl‐lithium, lithium naphthalene, or lithium metal.^[^ ^12^ ^]^ While many of the early studies on over‐lithiated cathode materials focused on the over‐lithiation of spinel oxides, such as Li_1+_ *_x_* Mn_2_O_4_ and Li_1+_ *_x_* Ni_0.5_Mn_1.5_O_4_,^[^ ^12b–e^ ^]^ recent works have also reported the possible over‐lithiation of SOTA LiNi*_x_* Mn*_y_* Co*_z_* O_2_ (NMC‐*xyz*, *x*+*y*+*z* = 1) layered oxides, yet precise control is required to avoid irreversible phase transitions.^[^ ^11^, ^12^, ^13^ ^]^ In both cases, either an additional production step is necessary or the use of an inert atmosphere is needed to handle the utilized chemicals and reaction conditions, raising safety concerns.

The incorporation of positive electrode pre‐lithiation additives within the positive electrode during processing is considered one of the most suitable scaling‐up strategies.^[^ ^7b^ ^]^ An ideal pre‐lithiation additive has to irreversibly release extra Li^+^ in the working potential range of the cathode active material, exhibit a high volumetric and gravimetric capacity, does not result in strong dead weight residues after use, and be only electrochemically active in the first charge of a LIB cell to compensate for ALL due to SEI formation at the anode surface.^[^ ^7a^ ^]^ Several lithium compounds have been investigated as cathode additives so far, including binary compounds, such as Li_2_S,^[^ ^14^ ^]^ Li_3_N,^[^ ^15^ ^]^ LiN_3_,^[^ ^16^ ^]^ Li_2_O,^[^ ^17^ ^]^ Li_2_O_2_,^[^ ^18^ ^]^ and LiF,^[^ ^19^ ^]^ as well as ternary compounds, such as Li_5_FeO_4_,^[^ ^20^ ^]^ Li_2_NiO_2_,^[^ ^21^ ^]^ Li_6_CoO_4_,^[^ ^22^ ^]^ and Li_2_MoO_3_.^[^ ^23^ ^]^ However, most of these additives are unstable in ambient atmosphere or incompatible with standard cathode processing protocols, e.g., using *N*‐methyl‐2‐pyrrolidone (NMP) as solvent.^[^ ^24^ ^]^ In addition, Li_2_O and Li_2_O_2_ are oxidized at high potentials >4.5 V versus Li|Li^+^ which can trigger oxidative decomposition of carbonate‐based electrolytes,^[^ ^25^ ^]^ dissolution of transition metals (TMs = Ni, Co and Mn) from NMC‐type layered oxide cathode materials and their subsequent deposition at the anode.^[^ ^25^, ^26^ ^]^

Organic lithium salts, such as lithium oxalate (Li_2_C_2_O_4_), squarate (Li_2_C_4_O_4_), and ketomalonate (Li_2_C_3_O_5_), have attracted attention as “sacrificial” pre‐lithiation additives for LIB cells and lithium ion capacitors.^[^ ^16^, ^27^ ^]^ Alternatively, sodium oxalate (Na_2_C_2_O_4_),^[^ ^28^ ^]^ squarate (Na_2_C_4_O_4_),^[^ ^29^ ^]^ ketomalonate (Na_2_C_3_O_5_),^[^ ^30^ ^]^ and rhodizonate (Na_2_C_6_O_6_)^[^ ^31^ ^]^ have been widely investigated to address the sodium deficiency issue in P2‐type sodium layered oxides. Although gravimetric capacities of such lithium salts only range between 400 and 600 mAh g^−1^,^[^ ^16^ ^]^ they can be handled safely in ambient atmosphere and have been reported to be oxidized at voltages below 4.5 V versus Li|Li^+^, with the only exception of Li_2_C_2_O_4_ which decomposes at ≈4.7 V versus Li|Li^+^. Solchenbach et al. reported an improved cycle life of LiNi_0.5_Mn_1.5_O_4_ (LNMO)||Si/graphite LIB cells containing 5 wt% Li_2_C_2_O_4_ due to the compensation of ALL induced by SEI formation and the beneficial impact of the released CO_2_ in effective SEI formation after additive decomposition ( = de‐lithiation),^[^ ^27a^ ^]^ in line with previous reports on CO_2_ as electrolyte additive.^[^ ^32^ ^]^ However, such high potentials (≈4.7 V *vs* Li|Li^+^) are incompatible with SOTA NMC‐type layered oxides due to enhanced aging mechanisms.^[^ ^27^, ^33^ ^]^ In contrast, Li_2_C_4_O_4_ is a low‐cost compound, stable in ambient atmosphere, compatible with industrial standard electrode processing protocols and has been reported to be oxidized in a suitable potential range from 3.5 to 4.5 V versus Li|Li^+^. According to Shanmukaraj et al., Li_2_C_4_O_4_ holds a good compromise between gravimetric capacity, decomposition potential and the quantity of gases released compared to Li_2_C_2_O_4_ and Li_2_C_3_O_5_.^[^ ^16^ ^]^ Several recent works by Arnaiz et al. proved the effectivity of Li_2_C_4_O_4_ as pre‐lithiation additive in lithium‐ion capacitors.^[^ ^27^, ^34^ ^]^ However, there are no detailed works reporting the influence of Li_2_C_4_O_4_ as positive electrode additive in the cycle life of Si‐based LIB cells to compensate ALL. Furthermore, an evaluation of the effect of decomposition products such as releasing gases and remaining cathode porosity on cell performance after oxidation of the additive is still lacking.

In this work, lithium squarate (Li_2_C_4_O_4_) is investigated for the first time as a pre‐lithiation additive for the cathode in lithium metal battery (LMB) cells and in LIB cells with a Si‐based negative electrode. As the oxidation potential of the additive might be influenced by its particle size, synthesis conditions of Li_2_C_4_O_4_ are first systematically adapted. The impact of the resulting morphologies and particle sizes are evaluated with respect to their electrochemical performance in Li_2_C_4_O_4_||Li metal cells. Second, different amounts of Li_2_C_4_O_4_ (0, 2.5, 5, and 10 wt%) are added to a NMC622 (LiNi_0.60_Co_0.20_Mn_0.20_O_2_) cathode following standard electrode processing routes. The rate capability and short‐term cycling performance of NMC622+Li_2_C_4_O_4_||Li metal cells are systematically evaluated regarding the amount of additive. Finally, the long‐term cycling stability of NMC622+Li_2_C_4_O_4_||Si/graphite LIB cells is investigated to determine whether pre‐lithiation with Li_2_C_4_O_4_ can compensate for ALL in the first cycle due to SEI formation and improve cycle life. *Post mortem* investigations of the surface of the positive and negative electrodes are performed by various analytical techniques to reveal morphological changes in the composite electrodes and differences in solid and cathode electrolyte interphases (SEI, CEI).

## 2 Results and Discussion

### 2.1 Li_2_C_4_O_4_ Characterization and Electrochemical Investigations in Li_2_C_4_O_4_||Li Metal Cells

Previous works have reported the need of reducing the particle size of pre‐lithiation cathode additives to promote complete decomposition at potentials <4.3 V (vs Li|Li^+^) due to the low electronic conductivity of some additives (e.g., Li_2_O,^[^ ^25^ ^]^ Li_2_O_2_,^[^ ^18^, ^26^ ^]^ and Na_2_C_4_O_4_^[^ ^28a^ ^]^). Particle size and morphology of pre‐lithiation additives are likely to impact the decomposition potential of the additive (i.e., lower decomposition potentials by decreasing the particle size) and the attainable charge capacity at a certain operating potential window (i.e., higher attainable capacity by decreasing the particle size). In this work, Li_2_C_4_O_4_ was easily synthesized from squaric acid (C_4_H_2_O_4_; 3,4‐dihydroxy‐3‐cyclobutene‐1,2‐dione) and lithium carbonate (Li_2_CO_3_) in a 1:1 molar ratio via a simple cation exchange.^[^ ^27b^ ^]^ Pure water (sample referred to as “Li_2_C_4_O_4_ H_2_O”) or a 1:1 water:ethanol mixture (sample referred to as “Li_2_C_4_O_4_ H_2_O:EtOH”) served as solvents (see the Experimental Section for further details). Ethanol (EtOH) was selected as antisolvent for crystallization to reduce the particle size due to the lower solubility of Li_2_C_4_O_4_ in organic solvents.^[^ ^28^, ^35^ ^]^ The mean particle diameter of Li_2_C_4_O_4_ was reduced from 16 ± 4 µm (Li_2_C_4_O_4_ H_2_O) to 7 ± 2 µm (Li_2_C_4_O_4_ H_2_O:EtOH) when using a H_2_O:EtOH mixture. In‐depth discussion on the characterization of synthetized Li_2_C_4_O_4_ powders and electrodes can be found in the Supporting Information, including scanning electron microscopy (SEM) images (Figure S1a,b, Supporting Information), particle size distribution (Figure S1c, Supporting Information), powder X‐ray diffraction (XRD; Figure S1d, Supporting Information), thermogravimetric analysis (Figure S2, Supporting Information), and electrochemical characterization in Li_2_C_4_O_4_||Li metal cells (Figure S3, Supporting Information), as well as analysis of the electrodes before and after cycling by XRD and SEM (Figure S4, Supporting Information). For electrochemical investigations of the additive alone, Li_2_C_4_O_4_ is irreversibly oxidized at voltages above ≈4.0 V in the first charge, as seen in Figure S3a (Supporting Information), exhibiting a sloping behavior according to the following disproportionation reaction^[^ ^27b^ ^]^

(1) $${\mathrm{Li}}_{2}C_{4}O_{4}\to 2{\mathrm{CO}}_{2}+2C+2e^{-}+2{\mathrm{Li}}^{+}$$

Compared to the theoretical gravimetric capacity of Li_2_C_4_O_4_ of 425 mAh g^−1^,^[^ ^27b^ ^]^ a maximum practical charge capacity of 404 ± 6 mAh g^−1^ is reached for the Li_2_C_4_O_4_ H_2_O:EtOH cells in the first cycle (30 wt% conductive carbon, see Figure S3b, Supporting Information). However, the amount of conductive agent within the electrode plays a critical role in the oxidation voltage of Li_2_C_4_O_4_, in agreement with previous works on Na_2_C_4_O_4_ as pre‐sodiation additive.^[^ ^28a^ ^]^ If not specified otherwise, Li_2_C_4_O_4_ H_2_O:EtOH was thus used for further investigations taking advantage of the lower decomposition voltage and slightly higher attainable first cycle specific charge capacities compared to Li_2_C_4_O_4_ H_2_O.

### 2.2 Impact of Li_2_C_4_O_4_ Addition on the Positive Electrode and Electrochemical Investigations in NMC622+Li_2_C_4_O_4_||Li Metal Cells

#### 2.2.1 Electrochemical Investigations in NMC622+Li_2_C_4_O_4_||Li Metal Cells

The influence of the Li_2_C_4_O_4_ addition on the electrochemical properties of the NMC622‐based cathodes was systematically evaluated regarding the additive content. Different amounts of Li_2_C_4_O_4_ (0, 2.5, 5, and 10 wt% based on the overall solid content) were added during processing of the NMC622‐based positive electrodes according to **Table** **1**. The weight ratio of active material:conductive agent:binder within the electrodes was kept constant at 94:03:03 once Li_2_C_4_O_4_ was fully oxidized. Due to the sufficient stability of the pre‐lithiation additive in air, the electrodes were processed at ambient conditions using the SOTA processing route with NMP as solvent and polyvinylidene difluoride (PVdF) as binder.

**Table 1:** Composition of the cathodes with respect to the pre‐lithiation additive content. The weight ratio of active material (NMC622), conductive agent (Super C65), and binder (PVdF) in the positive electrode is kept constant at 94:03:03, while 2.5, 5, or 10 wt% Li_2_C_4_O_4_ as additive is added

Li_2_C_4_O_4_ [wt%]	Active material NMC622 [wt%]	Conductive agent [wt%]	Binder [wt%]
0.00	94.00	3.00	3.00
2.50	91.65	2.93	2.93
5.00	89.30	2.85	2.85
10.00	84.60	2.70	2.70

**Figure 1:**
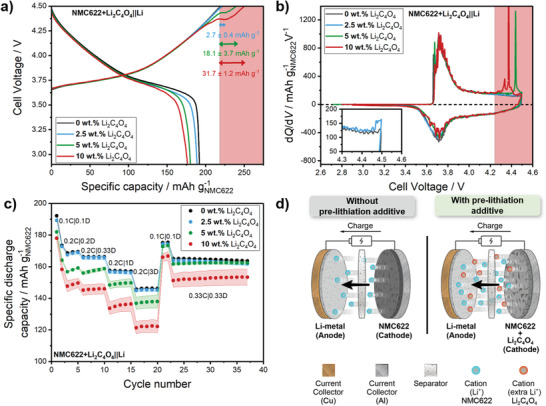
First cycle a) cell voltage profile and b) differential capacity (d*Q*/d*V*) versus voltage plots of NMC622+Li_2_C_4_O_4_||Li metal cells with or without additive at 0.1C from 2.9 to 4.5 V with an active mass loading of ≈5 mg_NMC622_ cm^−2^. The red dashed areas point out the range where Li_2_C_4_O_4_ is irreversibly oxidized. c) Rate capability of NMC622+Li_2_C_4_O_4_||Li metal cells with or without additive at different C‐rates from 0.1C to 3C at a cell voltage window between 2.9 and 4.3 V (1C = 170 mA g^−1^). The C‐rate was only varied upon discharge and kept at 0.2C during charge. Error bars represent the standard deviation of three cells tested for each content of additive. d) Schematic illustration of the pre‐lithiation approach with the help of positive electrode additives, providing an excess of lithium within the cell in the first charge cycle.

**Figure** **1**a,b shows the first cycle charge/discharge cell voltage profiles and differential capacity (d*Q*/d*V*) versus voltage plots of all NMC622+Li_2_C_4_O_4_||Li metal cells at 0.1C, respectively. As can be seen, Li_2_C_4_O_4_ is oxidized at higher voltages (>4.3 V) when combined with NMC622 using practical amounts of conductive carbon (3 wt%). Therefore, the first cycle upper cut‐off voltage was increased up to 4.5 V to promote the complete decomposition of Li_2_C_4_O_4_ without sacrificing cycle life and inducing faster aging phenomena. Consequently, the upper cut‐off charge voltage of the following cycles was set to 4.3 V. Short‐term cycling and rate capability electrochemical investigations of NMC622||Li metal cells without additive up to 4.3 and 4.5 V as first cycle upper cut‐off voltages can be found in Figure S5 (Supporting Information). As can be seen in Figure 1a, the first cycle charge capacity is gradually increased with the initial fraction of Li_2_C_4_O_4_ within the NMC622‐based cathode, providing an excess of active lithium in the first cycle. An additional voltage plateau growing in length with the amount of pre‐lithiation additive becomes apparent above 4.3 V. According to the theoretical capacity of Li_2_C_4_O_4_ (425 mAh g^−1^),^[^ ^27b^ ^]^ 2.5, 5, and 10 wt% of Li_2_C_4_O_4_ within the cathode should contribute to additional charge capacities of 10.6, 21.3, and 42.5 mAh g^−1^ when a complete oxidation is considered, respectively. Nevertheless, additional specific charge capacities of 2.7 ± 0.4 mAh g_NMC622_^−1^ (2.5 wt% Li_2_C_4_O_4_), 18.1 ± 3.7 mAh g_NMC622_^−1^ (5 wt% Li_2_C_4_O_4_), and 31.7 ± 1.2 mAh g_NMC622_^−1^ (10 wt% Li_2_C_4_O_4_) are reported. Therefore, Li_2_C_4_O_4_ is not fully oxidized <4.5 V in the first charge when considering practical amounts of conductive carbon, which however are necessary to minimize the use of inactive components and optimize the energy density of LIB cells.^[^ ^36^ ^]^

The decomposition of Li_2_C_4_O_4_ occurs at voltages >4.3 V, which is even better visualized by sharp peaks in the d*Q*/d*V* versus voltage plots shown in Figure 1b. However, the main oxidation voltage is shifted to lower values for higher initial amounts of Li_2_C_4_O_4_ within the cathode, i.e., the oxidation voltage was reduced to ≈4.35 V for the electrodes comprising 10 wt% Li_2_C_4_O_4_. In the electrode with only 2.5 wt% Li_2_C_4_O_4_, oxidation of the additive only starts shortly below 4.5 V (inset of Figure 1b), which might indicate why only 25% of the theoretical additional capacity is attained. When considering the same active material:conductive agent:binder ratio, one might have expected higher decomposition voltages for higher amount of additive due to the lower electrode conductivity. However, the opposite trend was always consistent throughout different experiments in both Li‐metal and Si‐based lithium ion cells. The reason for that cannot be proven but might be related to an increased ratio of Li_2_C_4_O_4_:conductive carbon, so that the first cycle decomposition at low rates (0.1C) can be improved by better electronic connection of the additive particles. Figure S3a (Supporting Information) also shows that a higher ratio of conductive additive toward Li_2_C_4_O_4_ will result in a decreased decomposition potential. The first cycle Coulombic efficiency (*C*_Eff_), calculated based on the first charge and discharge cycle capacities, is gradually decreased with increasing additive content from ≈87.6 (0 wt% Li_2_C_4_O_4_) to 70.9% (10 wt% Li_2_C_4_O_4_; see also Figure S6b, Supporting Information).

The short‐term cycle life and rate capability of NMC622+Li_2_C_4_O_4_||Li metal cells were systematically investigated with respect to the initial additive content within the cathode, to evaluate whether there is any detrimental impact in the electrochemical properties after the irreversible oxidation of Li_2_C_4_O_4_. Remarkably, although the first cycle attainable charge capacities gradually increase with the amount of additive (Figure 1a), the decrease in subsequent discharge capacities seems proportional to the additive content within this cell setup. The second cycle discharge capacities at a rate of 0.1C are 173.5 ± 0.4 mAh g_NMC622_^−1^ (0 wt% Li_2_C_4_O_4_), 172.7 ± 0.6 mAh g_NMC622_^−1^ (2.5 wt% Li_2_C_4_O_4_), 164.1 ± 0.9 mAh g_NMC622_^−1^ (5 wt% Li_2_C_4_O_4_), and 156.6 ± 0.2 mAh g_NMC622_^−1^ (10 wt% Li_2_C_4_O_4_). While cells with cathodes containing 2.5 wt% Li_2_C_4_O_4_ behave similarly to the reference cells, the rate capability of cells containing additive in significant amounts (5 and 10 wt%) is slightly worsened (Figure 1c). It is important to note that the pre‐lithiated cells show inferior discharge capacities to the reference cells until cycle ≈20^th^, considering the increasing discharge rate (up to 3C). From that cycle on, i.e., at decreased discharge rates (0.1C and 0.33C), capacities approach to those of the reference cells, except for those cells using 10 wt% Li_2_C_4_O_4_. Figure S6 (Supporting Information) confirms a similar impact on the rate capability when the cathode active material mass loading is increased from ≈5 to 10 mg_NMC622_ cm^−2^, and the mass loading might be even further increased to design high energy LIB cells.

Short‐term cycling performance shown in Figure S7a,b (Supporting Information) of cells with cathodes containing 5 and 10 wt% Li_2_C_4_O_4_ also reveals a drop in the attainable specific discharge capacities after formation cycles, in accordance with rate capability investigations. However, specific discharge capacities are then increasing over ≈40 cycles. Cells with electrodes containing 2.5 and 5 wt% of Li_2_C_4_O_4_ can outperform the reference cells in terms of capacities, whereas cells with positive electrodes containing 10 wt% Li_2_C_4_O_4_ cannot reach capacities comparable to the reference cells even after 100 cycles. The difference between the average charge and the average discharge voltages (Δ*V* calculated by *V*_average,charge_ − *V*_average,discharge_) over cycling is shown in Figure S7c (Supporting Information). Δ*V* can provide information about the polarization growth and impedance of a cell, i.e., higher Δ*V* values implicitly entail a higher cell impedance.^[^ ^37^ ^]^ As can be seen, cells containing Li_2_C_4_O_4_ show an enhanced polarization in comparison to the reference cells, but show a declining trend for the first ≈20–40 cycles.

Despite the fact that the addition of Li_2_C_4_O_4_ to the positive electrode in situ provides additional lithium in the cell in the first cycle (Figure 1d), which might be desirable to tackle the ALL challenge in Si‐based full‐cells, the addition of Li_2_C_4_O_4_ above a certain threshold level could indeed have a detrimental effect on the electrochemical behaviour of the cathode. The reported enhanced cell impedance and lower attainable discharge capacities might stem from other phenomena induced by the pre‐lithiation additive decomposition that negatively impact the subsequent electrochemical behaviour. One should also note that standard deviations between cells tend to increase with the amount of additive content. Thus, additive decomposition products introduce more uncertainties concerning cell performance.

#### 2.2.2 Post Mortem SEM and XPS Characterization of Cycled Positive Electrodes

After Li_2_C_4_O_4_ oxidation to gaseous CO_2_ according to Equation (1), changes in the electrode percolation network and an increase in porosity are expected to occur, along with a possible reaction of CO_2_ with the carbonate‐based electrolyte and further decomposition products at the cathode (and anode) surface. *Post mortem* analyses, including morphological investigations of cross sections of the cathode by SEM (i.e., to account for morphological changes within the electrode as a result of gas release after Li_2_C_4_O_4_ oxidation) and of the cathode electrolyte interphase (CEI, i.e., to account for compositional changes in the CEI) by X‐ray photoelectron spectroscopy (XPS), were performed to shed more light on the increased cell impedance and lower attainable specific discharge capacities after additive decomposition for the first cycles. For both experiments, NMC622+Li_2_C_4_O_4_||Li metal cells were cycled for one cycle in a cell voltage window of 2.9–4.5 V and electrodes were then retrieved from the cells in the discharged state for further analysis.

**Figure 2:**
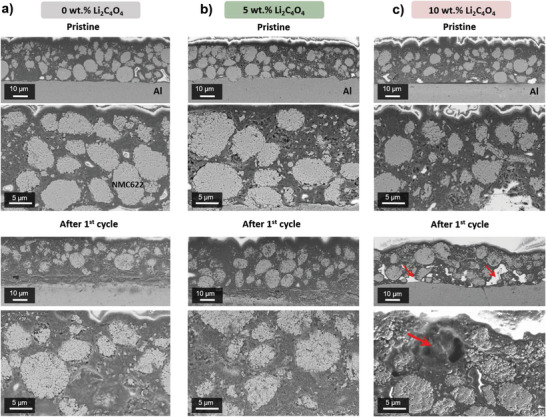
Cross‐sectional SEM images of pristine and cycled NMC622 electrodes comprising a) 0 wt%, b) 5 wt%, and c) 10 wt% Li_2_C_4_O_4_. Top images correspond to pristine electrodes, while bottom images correspond to cycled electrodes for one cycle (cell voltage range: 2.9–4.5 V in NMC622+Li_2_C_4_O_4_||Li metal cells). Red arrows point out emerging porosity after pre‐lithiation additive decomposition and the isolation of NMC622 active material particles.

**Figure** **2**a–c shows cross‐sectional SEM images of pristine and cycled positive electrodes originally containing 0, 5, and 10 wt% Li_2_C_4_O_4_, respectively. As can be seen, pristine electrodes show porosity in the sub‐micrometric range. On the contrary, cycled electrodes containing 10 wt% Li_2_C_4_O_4_ show porosity of larger scale (>5 µm), probably originating from the irreversibly decomposition of Li_2_C_4_O_4_ to CO_2_ and carbon, as it falls into the range of the mean particle diameter (7 ± 2 µm) reported in Figure S1c (Supporting Information). Therefore, changes in the electrode porosity after cycling become evident, leading to partially isolated NMC622 particles (Figure 2c) and the disruption to a certain extent of the 3D network of active material, conductive carbon, and binder. Besides morphological changes in the innermost parts of the electrode, the surfaces of the cycled electrodes comprising 5 and 10 wt% Li_2_C_4_O_4_ are wavier than the one of the reference electrode without additive (Figure 2a). This could be attributed to degassing of the pre‐lithiation additive, exerting pressure from the inside of the electrode. Even though no severe deconstruction of the cathodes is evident and the gases from the decomposition of the additive can be easily released after the formation cycle, the use of larger amounts (>10 wt%) of additive could be quite detrimental to the morphology of the initial electrode.

**Figure 3:**
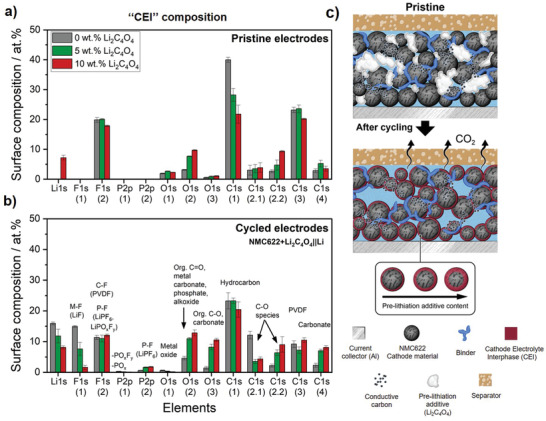
Relative atomic concentrations of the surface of the NMC622 electrodes a) before and b) after the first charge/discharge cycle in NMC622+Li_2_C_4_O_4_||Li metal cells (cell voltage range: 2.9–4.5 V) for three different Li_2_C_4_O_4_ concentrations within the electrodes (0, 5, and 10 wt%). Further details of the XPS fitting can be found in Figure S8 (Supporting Information). c) Schematic illustration of the positive electrode before and after cycling for one cycle showing the impact of pre‐lithiation additive decomposition on cathode morphology.

XPS investigations were carried out on pristine and cycled positive electrodes for one cycle containing 0, 5, and 10 wt% Li_2_C_4_O_4_ to get some insights into the elemental and chemical composition of the surface of the cathode. To ensure surface chemistry uniformity and reproducibility, XPS measurements were conducted to two electrodes per additive content (0, 5, and 10 wt%) and two spots per electrode. Estimated relative atomic concentrations of identified components at the surface of the different cathodes are shown in **Figure** **3**. Detailed C 1s, F 1s, O 1s, P 2p, Mn 2p, and Li 1s photoelectron core spectra can be found in Figure S8 (Supporting Information). It is important to note that XPS only provides information about the composition of the top surface of the cathode (limited penetration of ≈10 nm) and, therefore, about the elemental composition of topmost CEI surface layer but not about the whole electrode.

As can be seen in Figure 3b, the addition of Li_2_C_4_O_4_ to the positive electrode causes evident changes in the composition of the top surface layer. For cycled electrodes comprising 5 and 10 wt% Li_2_C_4_O_4_, C 1s spectra show an increase of oxidized species at the surface of the NMC622 electrode with increasing initial pre‐lithiation additive amount within the electrode, such as carbonates (e.g., Li_2_CO_3_) and C‐O species (Figure 3b and Figure S8a, Supporting Information).^[^ ^38^ ^]^ These findings are consistent with the evolution of the O 1s core spectra with respect to the pre‐lithiation additive content (Figure S8b, Supporting Information). In the O 1s spectra, three distinct peaks at ≈529.5, ≈532, and ≈533.8 eV can be observed for the pristine electrodes, which can be attributed to metal oxides of NMC622 (*M*‐O), organic C‐O and metal carbonates, and organic C‐O and organic carbonates, respectively.^[^ ^38^, ^39^ ^]^ After cycling, the *M*‐O signal abruptly vanishes with increasing Li_2_C_4_O_4_ amount. Moreover, there is an increased intensity at ≈533 eV in the O 1s core spectra, corresponding to carbonates, e.g., Li_2_CO_3_. These results further indicate participation of the decomposition products of Li_2_C_4_O_4_, such as CO_2_, in CEI formation.

The pristine positive electrodes show a distinct peak at ≈688.2 eV in the F 1s spectrum (Figure S8e, Supporting Information), which is related to the C‐F bonds of the PVdF binder used. After cycling for one cycle, the intensity of this peak is reduced for all electrodes along with a shift to lower binding energies due to the formation of additional species such as Li*_x_* PF*_y_* and/or LiPO*_x_* F*_y_* at the surface of the NMC electrode. Furthermore, F 1s core spectra indicate a decrease in the LiF signal at ≈685 eV for cycled electrodes comprising 5 and 10 wt% Li_2_C_4_O_4_, whereas P 2p spectra show only half of the typical electrolyte decomposition products of the conductive salt LiPF_6_, Li*_x_* PF*_y_*, and/or LiPO*_x_* F*_y_* (Figure 3a). The lower LiF signal does not necessarily mean that the amount of LiF in pre‐lithiated electrodes is lower than in the pristine electrodes. Instead, these findings can be related to a film covering the surface of the electrode and typical electrolyte decomposition products, in agreement with previous works on 5‐methyl‐1,3‐dioxolane‐2,4‐dione electrolyte additives in Si‐based LIB cells.^[^ ^38b^ ^]^ Schwenke et al. also reported that LiFePO_4_||graphite LIB cells filled with gaseous CO_2_ led to formation of Li_2_CO_3_ as the main additional component of the SEI and a suppression of typical electrolyte decomposition products.^[^ ^32a^ ^]^ The O 1s core spectra show a decrease in the signal of metal oxides with increasing additive content, which is in accordance with the Mn 2p core spectra and further confirms that the NMC622 active material is covered by a thick surface film after the first cycle. As can be seen in Figure S8c (Supporting Information), the Mn 2p content becomes negligible when adding 10 wt% Li_2_C_4_O_4_ to the positive electrode. This likely indicates that the decomposition of Li_2_C_4_O_4_ leads to a thicker CEI layer at the surface of the “pre‐lithiated” electrodes compared to the reference electrodes, which has been previously linked to a worse cell performance in NMC111||Li metal cells.^[^ ^40^ ^]^ However, it is important to note that the high reactivity of metallic Li suggests that results might only be valid for LMB cells and not for LIB cells.^[^ ^41^ ^]^ Therefore, further XPS experiments in Si‐based LIB cells will be discussed below.

In summary, the addition of Li_2_C_4_O_4_ additives greatly influences the surface composition of the NMC622 cathode. According to Figure 3, the decomposition of Li_2_C_4_O_4_ in the first charge step (= de‐lithiation of cathode) likely results in the formation of a thick CEI layer on the surface of the positive electrode, mainly composed by decomposition products of the pre‐lithiation additive (e.g., Li_2_CO_3_) caused by the release of CO_2_ gas. The CEI becomes thicker as the initial content of the pre‐lithiation additive within the electrode is increased (Figure 3b). The higher cell polarization and lower attainable capacities in the first cycles for NMC622+Li_2_C_4_O_4_||Li metal cells containing more Li_2_C_4_O_4_ additive (5 and 10 wt%) can thus be a combined result of emerging porosity within the electrode and a thick CEI at the cathode particles, which might impede the mobility of Li^+^ ions and result in increased interfacial resistances.^[^ ^40^ ^]^

### 2.3 Impact of Li_2_C_4_O_4_ Addition on Cycle Life of NMC622+Li_2_C_4_O_4_||Si/Graphite Cells

#### 2.3.1 Electrochemical Performance of NMC622+Li_2_C_4_O_4_||Si/Graphite Cells

The effect of different amounts of Li_2_C_4_O_4_ (0, 2.5, 5, and 10 wt%) on the electrochemical performance of NMC622+Li_2_C_4_O_4_||Si/graphite LIB cells was systematically investigated within the cell voltage range 2.8–4.2 V. A composite of Si nanowires and graphite with a specific capacity of ≈713 mAh g^−1^ (in Si/graphite||Li metal cells) was considered as the anode active material.^[^ ^42^ ^]^ To promote the almost complete decomposition of the pre‐lithiation additive, the first cycle upper cut‐off voltage was increased to 4.5 V with no significant detrimental effects on cycle life, as shown for cells without additive (Figure S9, Supporting Information).

The additional Li content provided by the pre‐lithiation additive in the first cycle has to be precisely adjusted in LIB full‐cells to avoid the risk of Li metal plating on top of the negative electrode surface, which might result in safety hazards during operation.^[^ ^43^ ^]^ Therefore, the negative/positive (N/P) capacity balancing ratio was optimized depending on the Li_2_C_4_O_4_ content by monitoring the potential evolution of the positive (NMC622) and negative (Si/graphite) electrodes in a three‐electrode setup using Li metal as reference electrode. In‐depth discussion on the N/P capacity balancing ratio can be found in the Supporting Information (Figure S10 and Table S1, Supporting Information). Cells were balanced by a N:P ratio of 1.20:1.00, with the only exception of the cells containing 10 wt% Li_2_C_4_O_4_ which were balanced to 1.25:1.00.

The long‐term stability was evaluated using different amounts of Li_2_C_4_O_4_ within the cathode in a two‐electrode cell configuration.^[^ ^44^ ^]^ After four formation cycles at 0.1C, long‐term cycling was investigated at 0.33C until reaching a state‐of‐health (SOH) of 60% (based on the fifth cycle specific discharge capacity). Systematic electrochemical investigations of the impact of different amounts of pre‐lithiation additive for the cathode on the cycle life of Si‐based full‐cells have rarely been reported in literature before. Only the works of Solchenbach et al.^[^ ^27a^ ^]^ for Li_2_C_2_O_4_ and Dose et al.^[^ ^20^ ^]^ for Li_5_FeO_4_ are known so far.

In the first charge/discharge cycles of a LIB cell, the amount of irreversible capacity can be directly correlated with the active lithium loss, as reported previously.^[^ ^5^, ^12^ ^]^ The amount of additional lithium needed to compensate for ALL due to SEI formation was thus calculated from the irreversible capacity in the first cycle of NMC622||Si/graphite LIB cell (0 wt% Li_2_C_4_O_4_), and was estimated to be ≈40 mAh g^−1^. Therefore, based on the theoretical discharge capacity of Li_2_C_4_O_4_, the amount of pre‐lithiation additive needed to fully compensate for ALL should be ≈9.4 wt%. However, it should be noted that complete decomposition of the additive is highly unlikely in this study due to the low amounts of conductive carbon considered. Consequently, the influence of different amounts from 2.5 to 10 wt% of Li_2_C_4_O_4_ within the electrode on prolonging cycle life was systematically evaluated.

**Figure 4:**
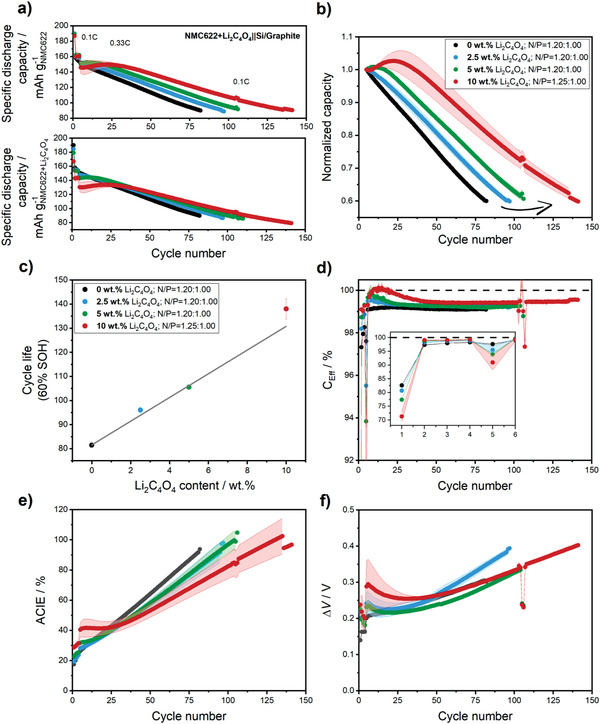
a) Specific capacity (calculated for the NMC622 and NMC622+Li_2_C_4_O_4_ mass loadings) and b) normalized capacity (calculated based on the fifth cycle discharge capacity) of the long‐term charge/discharge cycling experiments of NMC622+Li_2_C_4_O_4_||Si/graphite full‐cells at 0.33C (1C = 170 mA g^−1^); c) cycle numbers to 60% SOH against the initial content of Li_2_C_4_O_4_ within the cathode. Evolution of d) Coulombic efficiencies (*C*_Eff_), e) accumulated Coulombic inefficiencies (ACIE), and f) Δ*V* versus cycle number. The N/P ratio is 1.20:1.00, however, only for cells with the cathode containing 10 wt% Li_2_C_4_O_4_ it was set to 1.25:1.00. Cell voltage: 2.8–4.2 V (first cycle upper cut‐off voltage 4.5 V). Error bars represent the standard deviation of two cells.

**Figure** **4**a,b shows the evolution of the discharge capacities (based on the mass of NMC622 or NMC622 + Li_2_C_4_O_4_) and the normalized capacities calculated based on the fifth cycle discharge capacity versus cycle number until reaching 60% SOH. As can be seen, cells with initial amounts of 0, 2.5, 5, and 10 wt% Li_2_C_4_O_4_ in the cathode reach 60% SOH after ≈81, ≈96, ≈105, and ≈138 cycles, prolonging the cycle life by 19% (2.5 wt%), 30% (5 wt%), and 70% (10 wt%), respectively. Although first cycle charge specific capacities are gradually increasing with the initial amount of Li_2_C_4_O_4_ within the cathode (≈230 ± 1, 235 ± 3, 244 ± 1, and 262 ± 1 mAh g_NMC622_^−1^ for 0, 2.5, 5, and 10 wt% additive), first cycle discharge capacities are slightly lower for cells containing the additive (≈189.8 ± 0.3, 189 ± 2, 189.1 ± 1, and 187 ± 4 mAh g_NMC622_^−1^ for 0, 2.5, 5, and 10 wt% Li_2_C_4_O_4_). In this cell setup the difference in capacities in the first cycle is not so remarkable as in NMC622+Li_2_C_4_O_4_||Li metal cells, as the extra Li^+^ ions released by the oxidation of the additive in the first charge can compensate for active lithium losses due to SEI formation at the anode surface. When normalizing capacities to the mass of NMC622 plus Li_2_C_4_O_4_ (Figure 4a), discharge capacities of the pre‐lithiated cells are significantly lower than the reference cells. Although the additive is only electrochemically active in the first charge, the areal capacity is expected to be negatively affected due to the increased electrode porosity. The advantages of the presence of the additive, which is only electrochemically active in the first charge, only become apparent after ≈20–30 cycles for 5 and 10 wt% Li_2_C_4_O_4_ cells.

Figure 4c demonstrates a linear correlation between the cycle life to 60% SOH and the initial Li_2_C_4_O_4_ content within the cathode. When no lithium reservoir is present, cells suffer from an abrupt capacity fading and reach 60% SOH after only 82 cycles due to degradation phenomena caused by the use of ≈15 wt% Si within the anode, such as abrupt volume changes and continuous SEI (re‐)formation.^[^ ^3^ ^]^ In contrast, pre‐lithiated cells show a stable capacity retention for some cycles, depending on the initial amount of Li_2_C_4_O_4_ and thus the amount of active lithium available within the cell. When the active lithium reservoir provided by the additive is depleted, all pre‐lithiated cells suffer from a slope change of the discharge capacity decay. From that point onwards, there is a linear capacity fading upon cycling, similarly to the reference cells as the Li loss caused by ongoing parasitic side reactions cannot be further compensated. However, the slope in capacity fading is not so pronounced as for the reference cells. An abrupt capacity fading after lithium reservoir consumption has also been reported for electrochemical pre‐lithiation of Si‐based anodes by Overhoff et al.^[^ ^9c^ ^]^ The depletion of the lithium reservoir is one of the major limitations of pre‐lithiation approaches.^[^ ^9c^ ^]^ In general, positive effects on cycle life are observed by the addition of Li_2_C_4_O_4_, but the beneficial impact remains for less than 50 cycles for 10 wt% Li_2_C_4_O_4_.

The first cycle *C*_Eff_ (Figure 4d) decreases from 82.6% (0 wt% Li_2_C_4_O_4_) to 80.7%, 77.4%, and 71.3% for 2.5, 5, and 10 wt% Li_2_C_4_O_4_‐containing cells because of the higher initial attainable charge capacities due to the additive decomposition. If discharge capacities of cells containing additive are calculated based on the charge capacity of the reference cells without additive, first cycle *C*_Eff_ values of ≈82.5%, 82.3%, and 81.3% for 2.5, 5, and 10 wt% Li_2_C_4_O_4_‐containing cells are obtained, respectively. These small differences indicate that the occurring initial losses are not only affected by processes occurring at the anode, but also by irreversible reactions happening at the NMC active material.^[^ ^45^ ^]^ Due to kinetic limitations, the NMC material cannot reuptake all Li^+^ ions that are extracted during the initial charge. However, the *C*_Eff_ values are significantly improved for the pre‐lithiated cells for the first 20–50 cycles depending on the amount of additive originally present in the cathode compared to the reference cells (0 wt%). The *C*_Eff_ values in the fifth cycle are >99.5% when Li_2_C_4_O_4_ was added to the cells while the *C*_Eff_ of the reference cells is only ≈99.1%. Therefore, the pre‐lithiation additive can partially compensate for ALL, resulting in higher *C*_Eff_ and a more stable cycling performance. The depletion of the lithium reservoir can be observed by the declining trend of *C*_Eff_ from cycle 20 to 30 on. Despite this, the *C*_Eff_ values remain superior even after 100 cycles. While the *C*_Eff_ in cycle 80^th^ is 99.0% for the reference cells, cells containing 10 wt% Li_2_C_4_O_4_ show a *C*_Eff_ of ≈99.4%. This raises the question of whether the CO_2_ released by the Li_2_C_4_O_4_ additive can also have a beneficial effect on the electrochemical performance, similarly to previous findings by Solchenbach et al. in LNMO||Si/graphite LIB cells using Li_2_C_2_O_4_.^[^ ^27a^ ^]^ Future studies should shed more light on gas generation and the impact of gas release on cycle life in more realistic pouch cells.

For further comparison between the electrochemical performance of the different cells, the Coulombic inefficiencies for each cycle were calculated and accumulated over cycling (Figure 4e). Besides the cycling stability of the cells, the accumulated Coulombic inefficiencies (ACIEs) can reflect parasitic reactions within the LIB cell, i.e., increased ACIEs are an indication of enhanced parasitic side reactions.^[^ ^38^, ^46^ ^]^ Although ACIEs are significantly higher in the first charge/discharge cycles in the pre‐lithiated cells due to the lower initial *C*_Eff_ (higher attainable first charge capacities when using the pre‐lithiation additive), the ACIEs steadily increase for the first 20 cycles in the reference cells. The 10 wt% Li_2_C_4_O_4_‐containing cells show the lowest ACIE after ≈50 cycles. Moreover, Figure 4f shows the evolution of the Δ*V* versus the cycle number. As can be seen, cells containing the different amounts of pre‐lithiation additive show slightly increased polarization in the initial cycles which might be attributed to emerging porosity and impeded Li^+^ ions transport due to a thicker CEI. However, Δ*V* values decrease within the first ≈20 cycles, in agreement with previous results in Li metal cells. In the following cycles, pre‐lithiated cells show similar or lower polarization growth compared to the reference cells. To further confirm these observations, electrochemical impedance spectroscopy (EIS) experiments were carried out at 50% state‐of‐charge (SOC) after the first formation cycle. As can be seen in Figure S11 (Supporting Information), the charge transfer and surface film resistances of the cells monotonically increase with the initial amount of pre‐lithiation additive after irreversible oxidation. These results are in agreement with the trend of the Δ*V*, and can be further evidence that part of the released CO_2_ gases can contribute to form a thicker CEI and increase the overall interfacial resistances of the cell.

#### 2.3.2 Post Mortem XPS Characterization of Cycled Positive and Negative Electrodes

**Figure 5:**
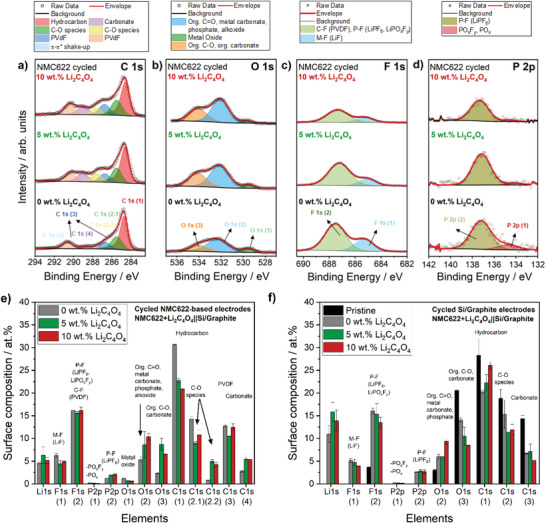
a) C 1s, b) O 1s, c) F 1s, and d) P 2p core spectra of cycled NMC622 positive electrodes comprising 0, 5, and 10 wt% Li_2_C_4_O_4_ after first charge/discharge cycle in NMC622+Li_2_C_4_O_4_||Si/graphite cells. Relative atomic concentrations of the surface of e) NMC622‐based positive electrodes and f) Si/graphite‐based negative electrode (cell voltage range: 2.8–4.5 V). Further details of the XPS fitting can be found in Figures S12 and S13 (Supporting Information).

To check whether the decomposition of Li_2_C_4_O_4_ influences not only the CEI composition, but also the SEI composition at the Si/graphite negative electrode surface, XPS analyses were performed on cycled electrodes for one cycle (voltage range: 2.8–4.5 V) in NMC622+Li_2_C_4_O_4_||Si/graphite LIB cells containing 0, 5, and 10 wt% Li_2_C_4_O_4_. C 1s, O 1s, F 1s, and P 2p photoelectron core spectra of the cycled NMC622 electrodes can be found in **Figure** **5**a–d. Further XPS core spectra can be found in Figures S12 and S13 (Supporting Information).

Based on the fitting of the core spectra,^[^ ^38a^ ^]^ the relative atomic concentrations of the surfaces of the different electrodes are calculated and shown in Figure 5. Results of the cathode are in good agreement with previous findings from NMC622+Li_2_C_4_O_4_||Li metal cells. However, changes in CEI composition are not as evident for increased amounts of Li_2_C_4_O_4_ possibly due to the high reactivity of metallic Li.^[^ ^41^ ^]^ The addition of Li_2_C_4_O_4_ apparently decreases the amount of fluorine species (LiF, LiPF_6_, and LiPO*_x_* F*_y_*; Figure 5c–e and Figure S13, Supporting Information) in the CEI/SEI layers, which might indicate less LiPF_6_ conductive salt decomposition products at the NMC622‐based and Si/graphite‐based electrode surfaces. In the O 1s spectra of the cathodes (Figure 5b), the content of metal oxides is reduced with increasing initial Li_2_C_4_O_4_ content within the cell, also suggesting a thicker CEI surface film at the cathode particles compared to the reference electrode. In addition, there is a slight decrease in the signal of the Mn 2p core spectra with increasing Li_2_C_4_O_4_ amount (Figure S12a, Supporting Information). The relative atomic concentrations of oxidized carbon species (organic C=O and C‐O) and carbonates are increased at the cathode surface (Figure 5a). While the high contribution of C‐O species in the O 1s spectra of the pre‐lithiated anodes may be related to the presence of significant amounts of Li_2_CO_3_,^[^ ^38b^ ^]^ there are no clear trends for the other contributions with respect to the additive content. The high content of C‐O species already present in the pristine electrode, along with the reduction of the electrolyte at the anode surface to form the SEI also forming C‐O species, prevents a proper differentiation of the exact role of the pre‐lithiation additive in SEI composition.

### 2.4 Implication of Li_2_C_4_O_4_ Addition on the Energy Density of NMC622+Li_2_C_4_O_4_|| Si/Graphite Cells

**Figure 6:**
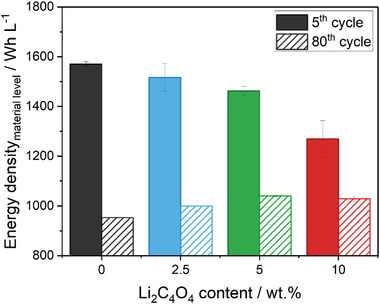
Energy densities of NMC622+Li_2_C_4_O_4_||Si/graphite full‐cells at 0.33C (1C = 170 mA g^−1^) based on the active mass of positive (including both NMC622 and Li_2_C_4_O_4_ weight) and negative active materials.

Results have proven that pre‐lithiated Si‐based LIB cells show an improved cycle life compared to the reference cells. While it is true that this approach can delay the capacity fading related to the use of Si in the negative electrode and compensate for initial ALL to a certain extent, it is also important to mention the potential shortcomings. The concept of pre‐lithiation with the help of positive electrode additives is easy to be scaled up, however, the emerging porosity or “dead volume” resulting from the decomposition of the additive has to be considered. Besides gravimetric capacity and cycle life, the energy density is a key performance indicator (KPI) for the practical application of LIB cells.^[^ ^36^ ^]^ As the emerging porosity created after the additive decomposition can have a detrimental impact on the volumetric capacity and thus on the energy density, **Figure** **6** shows the energy density on material level of the 5^th^ and 80^th^ cycles with different initial amounts of Li_2_C_4_O_4_ within the electrode. Owing to the difficulty of quantifying real changes in porosity and electrode volume change after irreversible oxidation of the pre‐lithiation additive, the energy density was calculated on material level rather than on electrode level. The energy density (E_v_) on material level was estimated from the mean discharge voltages (*U*_dis_), specific discharge capacities (*Q*_dis_), and the areal mass loading and the crystallographic density of active components (*ρ*_NMC622_ = 4.7 g cm^−3^,^[^ ^47^ ^]^ *ρ*_Li2C4O4_ = 1.9 g cm^−3^,^[^ ^48^ ^]^ and *ρ*_Si/graphite_ = 2.3 g cm^−3^) according to Betz et al.^[^ ^47^ ^]^

Although Li_2_C_4_O_4_ is only electrochemically active in the first charge, it was considered for all cycles, since the specific volume of electrode (including voids) per gram NMC622 is expected to increase. As can be seen, initial energy densities (5^th^ cycle) between 1200 and 1600 Wh L^−1^ are achieved at a rate of 0.33C. The initial energy density notably drops with increasing Li_2_C_4_O_4_. However, the beneficial effect of the pre‐lithiation approach becomes apparent after 80 cycles. While the cathode energy density of the reference cells drops to ≈953 Wh L^−1^, pre‐lithiated cells achieve values of ≈1000, 1041, and 1029 Wh L^−1^ for 2.5, 5, and 10 wt% Li_2_C_4_O_4_, respectively. These results highlight the limits of probably not only Li_2_C_4_O_4_, but many pre‐lithiation additives, that leave a “dead” volume (or weight for solid residues^[^ ^20^, ^22^ ^]^) within the electrode after irreversible oxidation and can adversely impact the energy density. Such negative impact on energy density may be avoidable if positive electrodes are calendered to lower initial porosities. However, the use of lower porosity values might induce particle cracking of cathode active particles.^[^ ^49^ ^]^

## 3 Conclusions

Within this work, lithium squarate (Li_2_C_4_O_4_) was evaluated as pre‐lithiation additive for the positive electrode to compensate for active lithium losses due to SEI formation at the Si‐based anode surface in LIB cells. Li_2_C_4_O_4_ holds great promise as additive due to its compatibility with industrial standard processing of the NMC‐based cathodes. The influence of different amounts of Li_2_C_4_O_4_ (0, 2.5, 5, and 10 wt%) within the cathode was systematically evaluated in NMC622+Li_2_C_4_O_4_||Li metal and NMC622+Li_2_C_4_O_4_||Si/graphite LIB cells. The additive was irreversibly de‐lithiated in the first charge cycle, increasing the attainable charge capacities and providing a surplus of active lithium within the cell. However, the discharge capacities were gradually decreased when increasing the amount of additive not only for the first cycle but, depending on the additive content, also for several initial cycles. *Post mortem* SEM investigations revealed an increased porosity and morphological changes of the electrodes because of the gas release when increasing the initial amount of pre‐lithiation additive. Moreover, XPS investigations clearly pointed out that the release of CO_2_ during the decomposition of the additive resulted in an abrupt increase in CEI thickness and a high occurrence of carbonates and C‐O species compared to the reference NMC622 cathodes. Nonetheless, the beneficial impact of pre‐lithiation on the electrochemical performance in terms of capacity retention when paired with Si/graphite anodes in LIB cells was successfully demonstrated. The cycle life to 60% SOH was linearly prolonged with the additive content within the cathode. Excess of active lithium provided by the additive in the first charge could compensate for active lithium losses due to continuous SEI (re‐)formation at the anode surface, resulting in an increased Coulombic efficiency for over 40 cycles when using 10 wt% Li_2_C_4_O_4_.

This specific approach of pre‐lithiation can compensate for active lithium losses of the initial charge/discharge cycles and is potentially suitable for industrial manufacturing processes. Unlike many other pre‐lithiation approaches that suffer from some safety issues, high reactivity, and poor homogeneity of pre‐lithiation (e.g., chemical pre‐lithiation by active reactants, “contact pre‐lithiation” by stabilized Li metal powder, etc.), pre‐lithiation additives can be easily added during the processing of the cathode paste, making no extra production step necessary. Despite the potential advantages, considerable challenges need to be overcome before this pre‐lithiation approach can be used in practical cells. The amount of additive must be carefully adjusted so as not to compromise the benefits provided in terms of cycle life. There might be a limit beyond which the additive might not be beneficial. The addition of higher amounts of additive (>10 wt%) would inevitably result in irreversible morphological changes of the electrode or Li metal plating in the first charge increasing safety risks. Moreover, the dead volume created after the additive is decomposed can adversely impact the cell energy density.

Future studies should therefore focus on investigating the interaction of the released gases with the electrolyte,the optimization of the additive amount and the CO_2_ gas formation during the formation cycles in large‐scale (e.g., pouch bag) cells to evaluate the feasibility of scaling‐up and prove relevance for practical applications. Finally, it may be interesting to note, that Li_2_C_4_O_4_ does serve as pre‐lithiation additive at the beginning of cycling, but that excess CO_2_, as side product after additive decomposition, can serve as electrolyte additive for the anode SEI during both, the formation cycles as well as during subsequent cycling.

## 4 Experimental Section

### Lithium Squarate (Li_2_C_4_O_4_) Synthesis

For the synthesis of Li_2_C_4_O_4_, two different approaches were addressed to adjust the particle size of the pre‐lithiation additive. First, 3,4‐dihydroxy‐3‐cyclobutene‐1,2‐dione (Sigma Aldrich, 99.0% purity) and lithium carbonate (Acros Organics, 99.0% purity) in a 1:1 molar mixture were dissolved in 150 mL of deionized water as previously reported.^[^ ^27b^ ^]^ Afterward, the deionized water solvent was evaporated at 60 °C under reduced pressure with a Büchi rotary evaporator. This material is referred to as “Li_2_C_4_O_4_ H_2_O” throughout the text.

In the second approach, 3,4‐dihydroxy‐3‐cyclobutene‐1,2‐dione (Sigma Aldrich, 99.0% purity) and lithium carbonate (Acros Organics, 99.0% purity) were dissolved in 200 mL of a 1:1 EtOH:de‐ionized water volume mixture. Afterward, the deionized water and EtOH were evaporated gradually under reduced pressure at 40 °C with a Büchi rotary evaporator. The resulting material is referred to as “Li_2_C_4_O_4_ H_2_O:EtOH”. The resulting white powders were ground in a mortar, further dried in a Büchi B‐585 glass drying oven under reduced pressure (<5 × 10^−2^ bar) at 50 °C for 12 h.

### Morphological and Structural Characterization of Li_2_C_4_O_4_ Powders and Li_2_C_4_O_4_ Containing Cathodes

Synthetized Li_2_C_4_O_4_ powders were evaluated by powder XRD (Bruker D8 Advance) using a Bragg‐Brentano geometry between 10° and 90° at a step size of 0.02° s^−1^, Cu‐K*α* radiation (*λ* = 0.154 nm) at 30 kV and 10 mA, and a divergence slit of 0.5 mm.

Surface morphologies of the synthetized Li_2_C_4_O_4_ powders and cathodes containing Li_2_C_4_O_4_ were investigated by SEM using a Carl Zeiss AURIGA field emission microscope with a Schottky field emitter as electron source and a typical accelerating voltage of 3 kV. The particle size distributions of the synthetized Li_2_C_4_O_4_ powders were analyzed by laser diffraction (CILAS 1064) using ethanol as solvent and few drops of dish soap as dispersant. Measurements were repeated three times to ensure a high reproducibility.

The thermal stability of Li_2_C_4_O_4_ cathode additive was investigated by thermogravimetric analysis (TGA) using a TGA Q5000‐IR (TA Instruments). Samples were loaded in an alumina crucible and measurements were performed under N_2_ flow at a heating rate of 10 K min^−1^ from room temperature to 600 °C.

### Electrode Preparation

For electrochemical investigations in Li_2_C_4_O_4_||Li metal cells, the positive electrode comprised 70 wt% (or 60 wt%) Li_2_C_4_O_4_ as active material, 20 wt% (or 30 wt%) carbon black as conductive agent (Super C65, Imerys Graphite & Carbon), and 10 wt% PVdF as binder (Solef 5130, Solvay). NMP (anhydrous, purity: 99.5%, Sigma‐Aldrich) was used as solvent, reaching a solid content of 20 wt%.

For electrochemical investigations in NMC622+Li_2_C_4_O_4_||Li metal and NMC622+Li_2_C_4_O_4_||Si/graphite cells, the positive electrodes consisted of 94 wt% NMC622 (LiNi_0.6_Mn_0.2_Co_0.2_O_2_, ShanShan Tech Co.; *d*_50_ = 10.22 µm, *d*_90_ = 14.09 µm) as active material, 3 wt% carbon black as conductive agent (Super C65, Imerys Graphite & Carbon), and 3 wt% PVdF as binder (Solef 5130, Solvay). If Li_2_C_4_O_4_ was added to the electrode paste (2.5, 5, or 10 wt% based on the total electrode paste solid weight), the proportions of the other components were reduced to maintain the same mass ratio between cathode active material, conductive agent, and binder (94:03:03) after Li_2_C_4_O_4_ decomposition in the first charge (according to Table 1). NMP (anhydrous, purity: 99.5%, Sigma‐Aldrich) was used as solvent, reaching a solid content of 50 wt%. For paste preparation of the cathode, powders were first dry‐mixed in a swing mill (15 min, 15 s^−1^, MM400, Retsch GmbH) followed by the addition of NMP (1 h, 30 s^−1^). After complete homogenization, the electrode paste was coated with a doctor‐blade (Zehntner GmbH) and an automatic film applicator (Sheen Instruments) on aluminum foil (20 µm, Nippon foil) which was previously washed with EtOH. The electrode sheets were then dried in an atmospheric oven at 80 °C for 2 h, punched out in 14 mm (for two‐electrode coin cells investigations) and 12 mm diameter discs (for three‐electrode Swagelok cell investigations), calendered (CLP 2025, Hohsen Corp.) to reach a porosity of 35% and dried in a Büchi B‐585 glass drying oven under reduced pressure (< 5 × 10^−2^ bar) at 120 °C for 12 h.

The average active mass loading of the positive electrode was ≈0.9 ± 0.3 mg_Li2C4O4_ cm^−2^ for Li_2_C_4_O_4_||Li metal cell investigations, resulting in areal capacities of ≈0.4 ± 0.1 mAh cm^−2^ based on the theoretical capacity of Li_2_C_4_O_4_ (425 mAh g^−1^).^[^ ^27b^ ^]^ For NMC622+Li_2_C_4_O_4_||Li metal cell and rate capability investigations, the average active material mass loading was ≈5.2 ± 0.8 and ≈9.6 ± 0.7 mg_NMC622 _cm^−2^. For NMC622+Li_2_C_4_O_4_||Si/graphite cell investigations the average active material mass loading was ≈5.0 ± 0.2 mg_NMC622_ cm^−2^, resulting in areal capacities of ≈0.84 ±0.03 mAh cm^−2^, based on the second cycle discharge capacity from NMC622||Li metal cells (cell voltage window 2.9–4.3 V).

The negative electrodes used for NMC622+Li_2_C_4_O_4_||Si/graphite full‐cell investigations comprised 85 wt% of a composite of Si nanowires and graphite (ENWIRES synthesized and supplied this composite sample as part of the project “SeNSE”; BET surface area = 16 m^2^ g^−1^; *d*_50_ = 17.7 µm, *d*_90_ = 21.5 µm) with a theoretical gravimetric capacity of ≈713 mAh g^−1^ and ≈15 wt% Si content as active material, 5 wt% carbon black as conductive agent (Super C65, Imerys Graphite & Carbon), 7.7 wt% sodium‐carboxymethyl cellulose (Na‐CMC, Walocel CRT 2000 PPA12, Dow Wolff Cellulosics), and 2.3 wt% polyacrylic acid (PAA, average Mw 450 000, Sigma‐Aldrich) as binders. Deionized water was used as solvent. For paste preparation, the binders and 1.2 wt% (referring to the overall solid weight) lithium hydroxide (98%, Fisher Chemical) were dissolved in ≈60 wt% of the deionized water in a planetary centrifugal mixer (20 min, 1700 rpm, ARM‐310CE, Thinky Corporation). Afterward, conductive agent, active material, and the other ≈40 wt% of the deionized water were added. The electrode paste was then homogenized by a planetary centrifugal mixer (ARM‐310CE, Thinky Corporation) at a speed of 1700 rpm for 15 min and 500–700 rpm for 5 min. The anode paste was coated on smooth copper foil (10 µm, Nippon foil) previously washed with EtOH. After pre‐drying at 60 °C in an atmospheric oven for 2 h, the sheets were dried in an oven at 120 °C for 10 h under reduced pressure. Circular electrode disks with Ø = 15 mm (for coin cells investigations) and Ø = 12 mm (for Swagelok cells investigations) in diameter were punched out. The average active mass loading of the anodes was ≈1.43 ± 0.03 mg cm^−2^, resulting in an areal capacity of ≈1.02 ± 0.02 mAh cm^−2^ based on the practical capacity of the Si/graphite composite (≈713 mAh g^−1^) obtained from the first cycle charge capacity from Si/graphite||Li metal cell investigations (cell voltage window between 0.01 and 1.5 V).

### Cell Assembly and Electrochemical Characterization

Electrochemical investigations were conducted in two‐electrode configuration in coin cells (CR2032, Hohsen Corporation) assembled in a dry room with a dew point of at least −50 °C (relative humidity of 0.16%).^[^ ^44^ ^]^ A polymer membrane (Ø = 16 mm, 2 layers, Celgard 2500, Celgard) served as a separator and was soaked with 70 µL of the electrolyte LP57 (1 m LiPF_6_ in 3:7 vol% ethylene carbonate/ethyl methyl carbonate, EC/EMC, Solvionic) with 10 wt% fluoroethylene carbonate (FEC, 10 wt%) as SEI‐forming additive.^[^ ^50^ ^]^ At least two to three cells per sample were assembled to ensure reproducible results. The standard deviation between different cells is represented as error bars in the corresponding figures.

For Li_2_C_4_O_4_||Li metal cells and NMC622+Li_2_C_4_O_4_||Li metal cells, Li_2_C_4_O_4_ (Ø = 14 mm), NMC622 (Ø = 14 mm), and Si/graphite (Ø = 15 mm) composite electrode disks were used as positive electrode and Li metal as negative electrode (Ø = 15 mm), lithium metal foil, 500 µm in thickness; battery grade: purity ≥99.9%, China Energy Lithium (CEL Co.). For NMC622+Li_2_C_4_O_4_||Si/graphite investigations, NMC622‐based electrodes (Ø = 14 mm) as positive electrode and Si/graphite‐based (Ø = 15 mm) electrodes as negative electrode were used. The negative/positive (N/P) capacity balancing ratio was set to 1.20:1.00 if not indicated otherwise to avoid Li metal plating at the Si/graphite negative electrode, based on the first cycle areal capacities of the positive (≈0.84 ± 0.03 mAh cm^−2^; cell voltage window 2.9–4.3 V) and negative electrodes (≈1.02 ± 0.02 mAh cm^−2^; cell voltage window between 0.01 and 1.5 V) in NMC622+ Li_2_C_4_O_4_||Li metal and Si/graphite||Li metal cells.

Further NMC622+Li_2_C_4_O_4_||Si/graphite full‐cell electrochemical investigations were carried out in a three‐electrode configuration in T‐cells (Swagelok, custom in‐house design) to determine the evolution of individual potentials of both positive and negative electrodes upon cycling and proper N/P balancing ratio to prevent Li metal plating. The body of the Swagelok cells was previously covered with an insulating Mylar foil (polyethylene terephthalate, PET). Three‐layered polyolefin Freudenberg FS2190 disks were used as separators (Freudenberg) with a size of Ø = 12 mm and Ø = 8 mm soaked with 90 and 60 µL electrolyte, respectively. NMC622 (Ø = 12 mm) and Si/graphite (Ø = 12 mm) electrodes and Li metal (Ø = 8 mm) were used as positive, negative and reference electrodes, respectively.

Constant current (CC) charge–discharge cycling was performed on a Maccor Series 4000 battery tester (Maccor, Inc.) at 20 °C. The specific current for a rate of 1C was defined as 440 mA g^−1^ for Li_2_C_4_O_4_||Li metal cells, 700 mA g^−1^ for Si/graphite||Li metal cells, and 170 mA g^‐1^ for NMC622+Li_2_C_4_O_4_||Li metal and NMC622+Li_2_C_4_O_4_||Si/graphite cells. The rate capability of the NMC622‐based positive electrodes with or without the pre‐lithiation additive was investigated in two‐electrode configuration NMC622||Li metal cells, according to the following cycling procedure: 6 h at open‐circuit‐voltage (OCV) followed by two formation cycles at 0.1C, three cycles at 0.2C, and five cycles at 0.33C, 0.5C, 1C, and 3C each, two cycles at 0.1C, and 15 cycles at 0.33C. The C‐rate was only varied upon discharge and kept at 0.2C during charge to minimize inhomogeneous Li metal plating on the Li metal anode. Rate capability investigations of Li metal cells were conducted in a cell voltage window between 2.9 and 4.3 V, only in the first cycle the upper cut‐off voltage was increased up to 4.5 V to promote additive decomposition.

The long‐term cycle life with respect to the pre‐lithiation additive amount (0, 2.5, 5, and 10 wt%) was also evaluated in NMC622+Li_2_C_4_O_4_||Li metal cells within the cell voltage range of 2.9‒4.3 V and in NMC622+Li_2_C_4_O_4_||Si/graphite full‐cells within the range of 2.8‒4.2 V. The first cycle upper cut‐off voltage was set to 4.5 V for both cell setups if not indicated otherwise. NMC622+Li_2_C_4_O_4_||Si/graphite full‐cells were charged to 1.5 V for 15 min prior to 6 h at OCV to prevent Cu current collector dissolution.^[^ ^51^ ^]^ Cells were then cycled for four cycles at 0.1C for interphase formation, followed by cycling at 0.33C for 100 cycles (NMC622+Li_2_C_4_O_4_||Li metal) or until reaching 60% SOH (NMC622+Li_2_C_4_O_4_||Si/graphite), respectively. Each 100^th^ cycle, cells were cycled at 0.1C again for two cycles to evaluate the capacity retention. After each charge step, a constant voltage (CV) step was performed until the specific current reached values below 0.05C.

EIS measurements were performed on a potentiostat/galvanostat VMP3 (BiologicScience Instruments) using NMC622+Li_2_C_4_O_4_||Si/graphite full‐cells in a two‐electrode configuration. After a 6 h OCV step, the cells were charged for one cycle at 0.1C according to the constant current charge/discharge protocol described above. EIS was measured at a discharge voltage of 3.5 V. The spectra were obtained at an amplitude of 5 mV in a frequency range from 0.1 MHz to 0.1 Hz.

### Post Mortem Characterization

For post mortem morphological analysis of the cycled electrodes, cells were disassembled in the discharged state at 2.8 V (NMC622+Li_2_C_4_O_4_||Si/graphite cells) or 2.9 V (NMC622+Li_2_C_4_O_4_||Li metal or Li_2_C_4_O_4_||Li metal cells) after the first cycle. Harvested electrodes were rinsed with 200 µL dimethyl carbonate (DMC) to remove salt impurities. To evaluate porosity changes, cross‐sections of cycled electrodes (after the first cycle) were prepared by gluing the electrodes in a multi‐clip on the bottom of a teflon cup which was then filled with a mixture of epoxy resin and hardener (Epofix) in a weight ratio of 25:3 and evacuated in vacuum (CitoVac) to prevent air entrapment. The surface was polished with a polishing machine (Tegramin, Struers) applying anhydrous lubricant and diamond containing abrasives with a decreasing particle size in every polishing step. Finally, the samples were washed carefully with ethanol and air‐dried.

Investigations of the SEI and CEI were conducted by X‐ray photoelectron spectroscopy using an Axis Ultra DLD XPS (Kratos Analytical) with a monochromatic Al K*α* source (*hν* = 1486.6 eV) at an emission current of 10 mA with an accelerating voltage of 12 kV. Positive charging of the sample was suppressed by using a charge neutralizer. Investigated cells were disassembled in the discharged state after the first cycle (i) NMC622+Li_2_C_4_O_4_||Si/graphite cells; voltage window 2.8–4.5 V; (ii) NMC622+Li_2_C_4_O_4_||Li metal cells; voltage window 2.9–4.5 V) in an argon‐filled glove box to avoid contact with atmospheric air. For reproducibility, two electrodes per additive content (0, 5, and 10 wt%) and two spots per electrode were measured. Data were analyzed by CasaXPS (v2.3.23, Casa Software) and referenced to the hydrocarbon signal C 1s corresponding to 284.8 eV.

XRD measurements were performed to composite positive electrodes before cycling (pristine electrodes) and after the first cycle to evaluate the reversibility of the decomposition of Li_2_C_4_O_4_ in the first charge/discharge cycles of Li_2_C_4_O_4_||Li metal cells cycled within the cell voltage range of 2.9–4.3 V.

## Conflict of Interest

The authors declare no conflict of interest.

## Supporting information

Supporting Information

## Data Availability

The data that support the findings of this study are available from the corresponding author upon reasonable request.
