# Supplementary material for: Opportunities and Challenges of Li2C4O4 as Pre‐Lithiation Additive for the Positive Electrode in NMC622||Silicon/Graphite Lithium Ion Cells
Source: Adv Sci (Weinh). 2022 Jul 7;9(24):2201742. doi: 10.1002/advs.202201742 (PMC9403639; doi:10.1002/advs.202201742)
Supplement: Supplementary file 1 — Supporting Information [file ADVS-9-2201742-s001.pdf]

ADVANCED SCIENCE
Open Access

Supporting Information

for *Adv. Sci.*, DOI 10.1002/advs.202201742

Opportunities and Challenges of $Li_2C_4O_4$ as Pre-Lithiation Additive for the Positive Electrode in NMC622||Silicon/Graphite Lithium Ion Cells

Aurora Gomez-Martin, Maike Michelle Gnutzmann, Egy Adhitama, Lars Frankenstein, Bastian Heidrich, Martin Winter and Tobias Placke**

Supporting Information

Opportunities and Challenges of $Li_2C_4O_4$ as Pre-Lithiation Additive for the Positive Electrode in NMC622||Silicon/Graphite Lithium Ion Cells

Aurora Gomez-Martin[1,], Maike Michelle Gnutzmann[1,2], Egy Adhitama[1,2], Lars Frankenstein[1], Bastian Heidrich [1], Martin Winter[1,4] and Tobias Placke[1, *]*

Dr. Aurora Gomez-Martin, Maike Michelle Gnutzmann, Egy Adhitama, Lars Frankenstein, Bastian Heidrich, Prof. Dr. Martin Winter and Dr. Tobias Placke

[1] University of Münster, MEET Battery Research Center, Institute of Physical Chemistry

Corrensstr. 46, 48149 Münster, Germany

*E-mail: agomezma@uni-muenster.de (A.G-M), tobias.placke@uni-muenster.de (T.P)

Maike Michelle Gnutzmann, Egy Adhitama

[2] University of Münster, International Graduate School for Battery Chemistry, Characterization, Analysis, Recycling and Application (BACCARA)

Corrensstr. 40, 48149 Münster, Germany

Prof. Dr. Martin Winter

[4] Helmholtz-Institute Münster, IEK-12, Forschungszentrum Jülich GmbH

Corrensstr. 46, 48149 Münster, Germany

Synthesis of $Li_2C_4O_4$ and electrochemical characterization in $Li_2C_4O_4$||Li metal cells

Scanning electron microscopy (SEM) images shown in **Figure S1a** and **b** reveal significant differences in particle size of the $Li_2C_4O_4$ powdered materials depending on the synthesis conditions. Both materials have an irregular morphology, with large particles formed by the agglomeration of nano-sized primary particles (≈50-500 nm). The sample $Li_2C_4O_4$ H_2O:EtOH shows a significant reduction in particle size and a lower degree of agglomeration between individual grains. Particle size distributions shown in **Figure S1c** confirm that the mean particle diameter of $Li_2C_4O_4$ H_2O is 16 ± 4 µm, while ≈90% of the particles (d_{90}) exhibit a diameter below 35 ± 7 µm. The mean particle size and d_{90} values were significantly reduced to 7 ± 2 µm and 19 ± 5 µm for the $Li_2C_4O_4$ H_2O:EtOH sample, respectively.

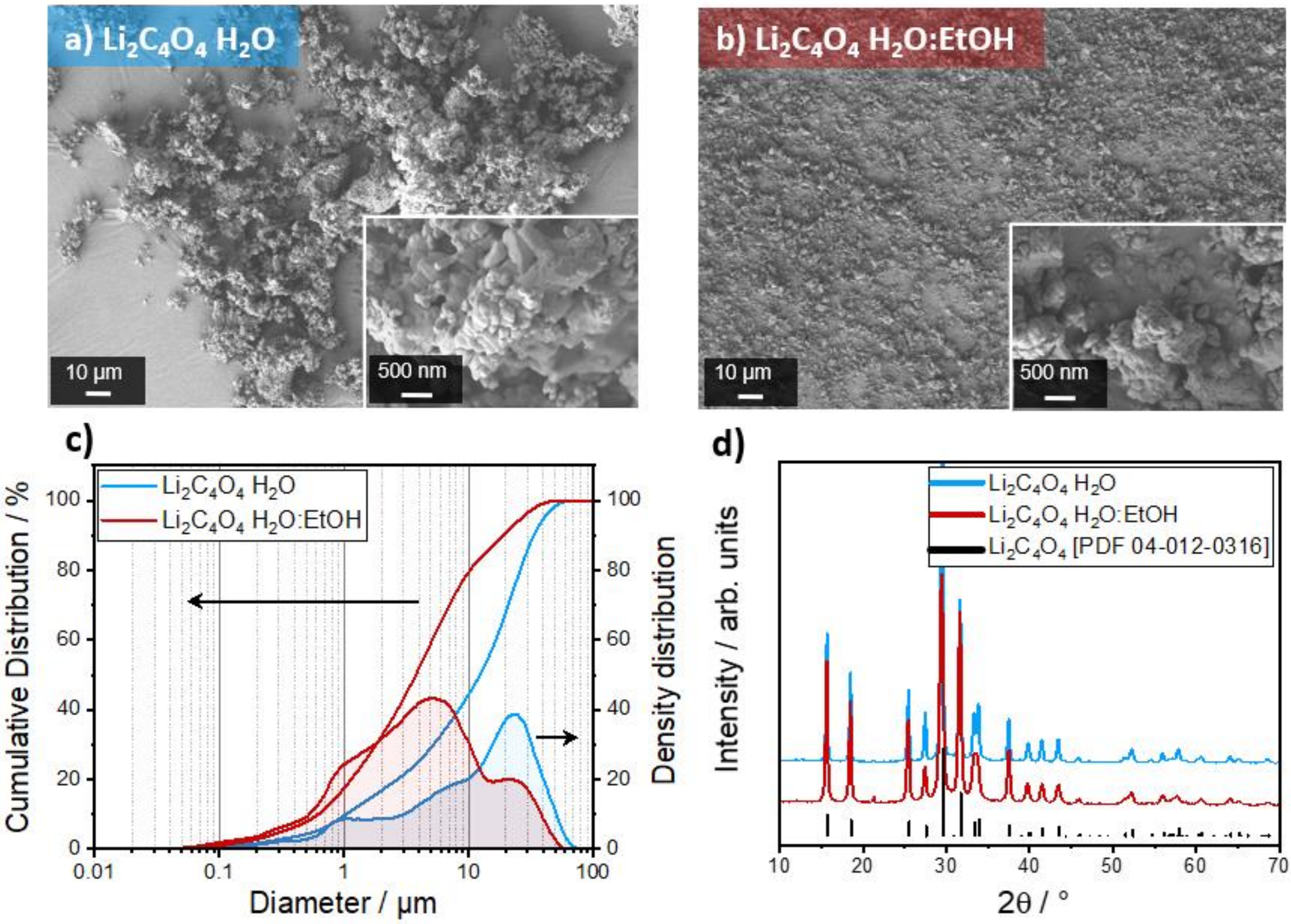

Figure S1. SEM micrographs of synthetized a) "$Li_2C_4O_4$ H_2O" and b) "$Li_2C_4O_4$ H_2O:EtOH" samples at different magnifications. High-magnification images are shown in the inset of the figures; c) Particle size distribution and d) XRD patterns of "$Li_2C_4O_4$ H_2O" and "$Li_2C_4O_4$ H_2O:EtOH" powdered samples.

Powder X-ray diffraction (XRD) patterns for the different materials shown in **Figure S1d** confirm the successful synthesis of $Li_2C_4O_4$. Main reflections can be indexed to a monoclinic phase belonging to the space group C2/m (PDF 04-012-0316). The sample $Li_2C_4O_4$ H_2O:EtOH exhibits a clear broadening of the reflections in the XRD pattern indicating a decrease in crystallinity and crystallite size when using EtOH as antisolvent for crystallization. As drying at above 100 °C is a standard processing step for positive composite electrodes for LIBs, the thermal stability of $Li_2C_4O_4$ was investigated by thermogravimetric analysis (TGA) under inert atmosphere from room temperature up to 600 ºC. As shown in **Figure S2**, $Li_2C_4O_4$ is thermally stable up to ≈430 °C.

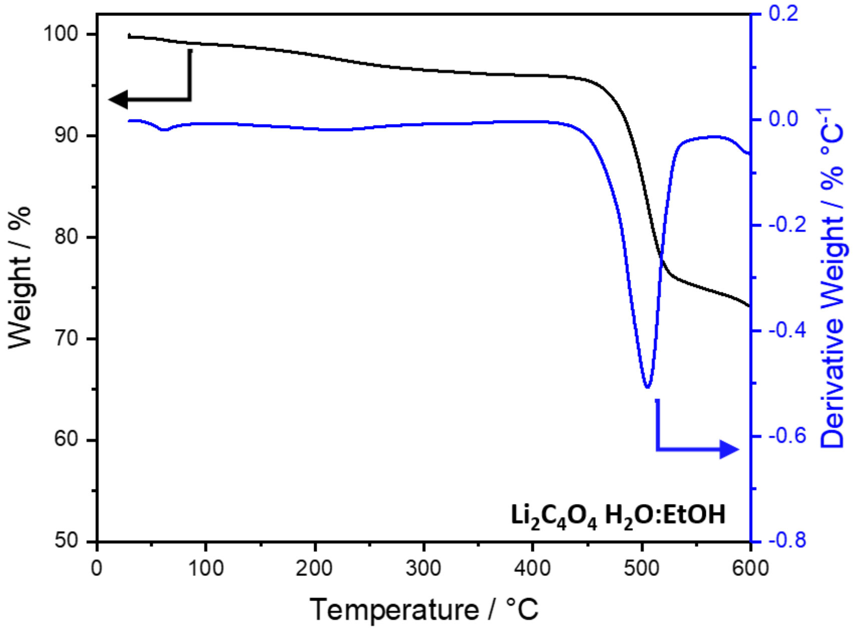

Figure S2. Thermogravimetric analysis (TGA, left axis) and differential thermal analysis (DTA, right axis) of $Li_2C_4O_4$ H_2O:EtOH powdered sample.

Electrochemical properties of the $Li_2C_4O_4$ pre-lithiation additives were first evaluated in $Li_2C_4O_4$||Li metal cells in the standard operating voltage window of NMC-type layered oxides (2.8 to 4.3 V).[1] **Figure S3a** and **b** show the first cycle charge/discharge voltage profiles at 0.1C and the evolution of the specific capacity *vs.* cycle number, respectively. For these experiments, electrodes comprising 70 wt.% (or 60 wt.%) $Li_2C_4O_4$ as active material and 20 wt.% (or 30 wt.%) carbon black as conductive agent (CA, Super C65) were tested. Though such high amounts of CA are not realistic for practical LIB electrodes, these electrode compositions were considered to gain some insight into the different electrochemical properties of the pre-lithiation additives depending on the mean particle sizes. As can be seen in **Figure S3a**, $Li_2C_4O_4$ is irreversibly oxidized at voltages above ≈4.0 V, exhibiting a sloping behaviour according to the following disproportionation reaction:[2]

$$Li_2C_4O_4 \rightarrow 2\ CO_2 + 2\ C + 2\ e^- + 2\ Li^+ \quad (1)$$

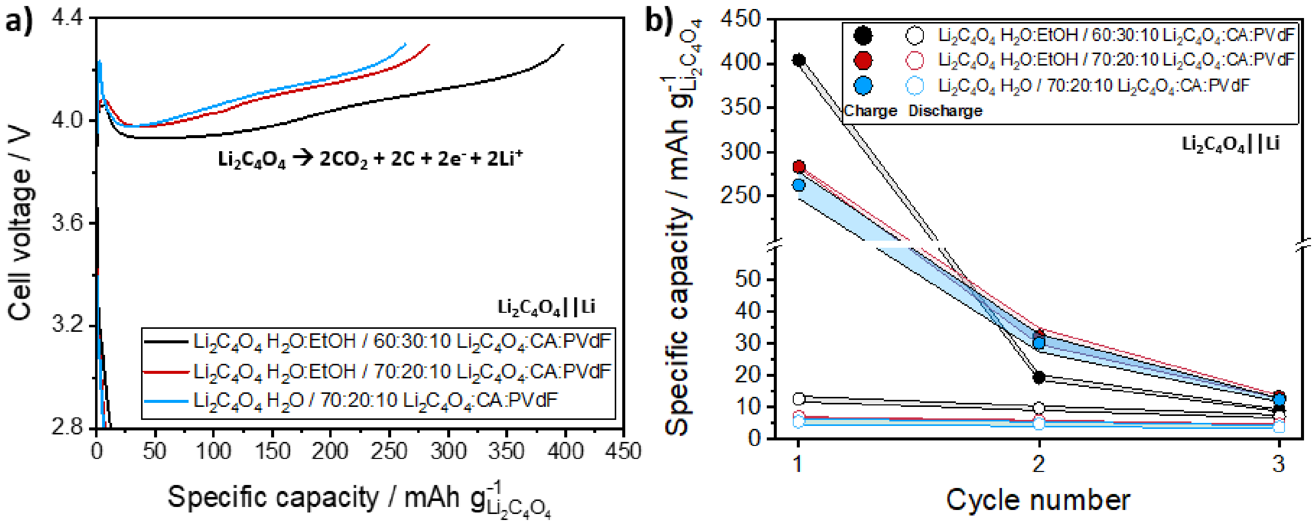

Figure S3. a) First cycle voltage profiles and b) specific charge and discharge capacities *vs.* cycle number of $Li_2C_4O_4$||Li metal cells (two-electrode configuration) cycled at 0.1C from 2.8 to 4.3 V (1C = 440 mA g^{-1}). The active material $Li_2C_4O_4$ corresponds to 70 or 60 wt.% of the electrode mass, the conductive agent (CA) to 20 or 30 wt.%, respectively, and the binder (PVdF) to 10 wt.%.

However, significant overvoltages lead to an initial decomposition voltage > 4.2 V for $Li_2C_4O_4$ H_2O. Changes in the onset of the decomposition voltage for the different materials can be related to the different particle sizes. By decreasing the mean particle size for the $Li_2C_4O_4$ H_2O:EtOH sample, the onset of the oxidation voltage is lowered by more than 0.1 V. Cells with electrodes comprising 70 wt.% of the differently synthesized $Li_2C_4O_4$ as active materials reach specific charge capacities of 263 ± 15 mAh g^{-1} ($Li_2C_4O_4$ H_2O) and 284 ± 1 mAh g^{-1} ($Li_2C_4O_4$ H_2O:EtOH), which are far away from the theoretical value of 425 mAh g^{-1}.[2] Upon lowering the active material content from 70 to 60 wt.% and increasing the CA content from 20 to 30 wt.%, cells containing $Li_2C_4O_4$ H_2O:EtOH can reach a specific charge capacity of 404 ± 6 mAh g^{-1} in the first cycle. Therefore, the amount of conductive agent within the electrode might play a critical role in the oxidation voltage of $Li_2C_4O_4$, in agreement with previous works on $Na_2C_4O_4$ as pre-sodiation additive in sodium ion batteries.[3] As can be seen in **Figure S3b**, there is almost no electrochemical activity in the first discharge cycle, and charge capacities are gradually decreasing in subsequent cycles. Discharge capacities, amounting to a total of ≈29 ± 1 mAh g^{-1} in the first three cycles for "$Li_2C_4O_4$ H_2O:EtOH", can be attributed to the electrochemical activity of the CA agent in $LiPF_6$-based organic electrolytes.[4] SEM and XRD analyses of pristine and cycled electrodes (**Figure S4**) confirm the complete irreversible oxidation of $Li_2C_4O_4$ after three charge/discharge cycles when the CA content within the electrode is 30 wt.%, leaving behind homogeneous porosity once the additive is irreversibly oxidized most likely to CO_2

and carbon.[2] In contrast, some remaining $Li_2C_4O_4$ particles can be found in electrodes containing 20 wt.% of CA. If not specified otherwise, $Li_2C_4O_4$ H_2O:EtOH was thus used for further investigations taking advantage of the lower decomposition voltage and slightly higher attainable first cycle specific charge capacities.

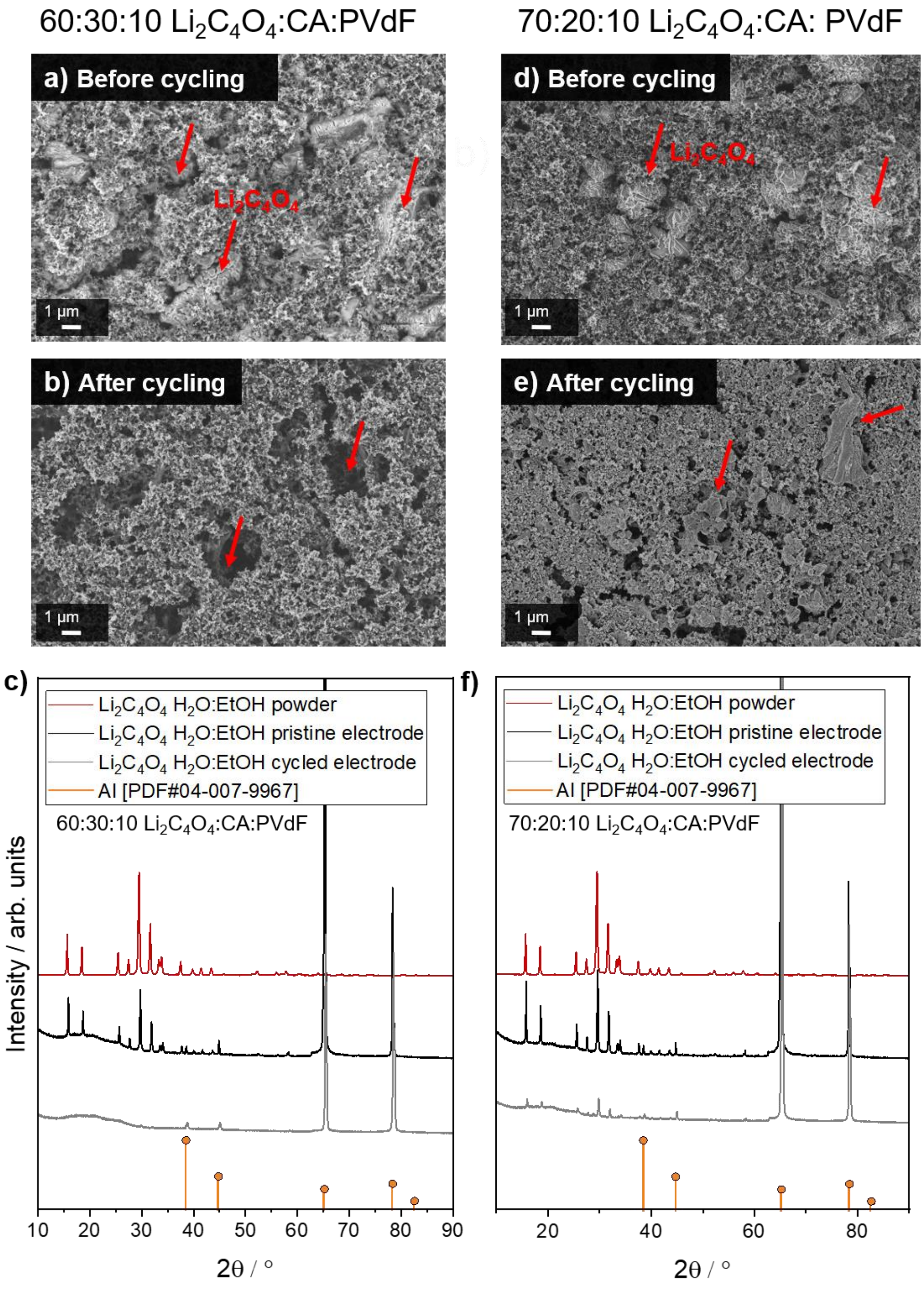

Figure S4. SEM micrographs of positive electrodes with (a, b) 60 wt.% or (d, e) 70 wt.% lithium squarate ($Li_2C_4O_4$ H_2O:EtOH), (a, b) 30 wt.% or (d, e) 20 wt. % carbon black (Super C65) as conductive agent (CA) and 10 wt.% PVdF as binder (a, d) before and (b, e) after three cycles at 0.1C in $Li_2C_4O_4$||Li metal cells. Red arrows point out $Li_2C_4O_4$ particles or arising pores; (c, f) XRD patterns of $Li_2C_4O_4$ powder ($Li_2C_4O_4$

H_2O:EtOH) and of $Li_2C_4O_4$-based electrodes before and after three cycles between 2.8 V and 4.3 V (1C = 440 mA g^{-1}) .

Electrochemical characterization of NMC622+$Li_2C_4O_4$||Li metal cells

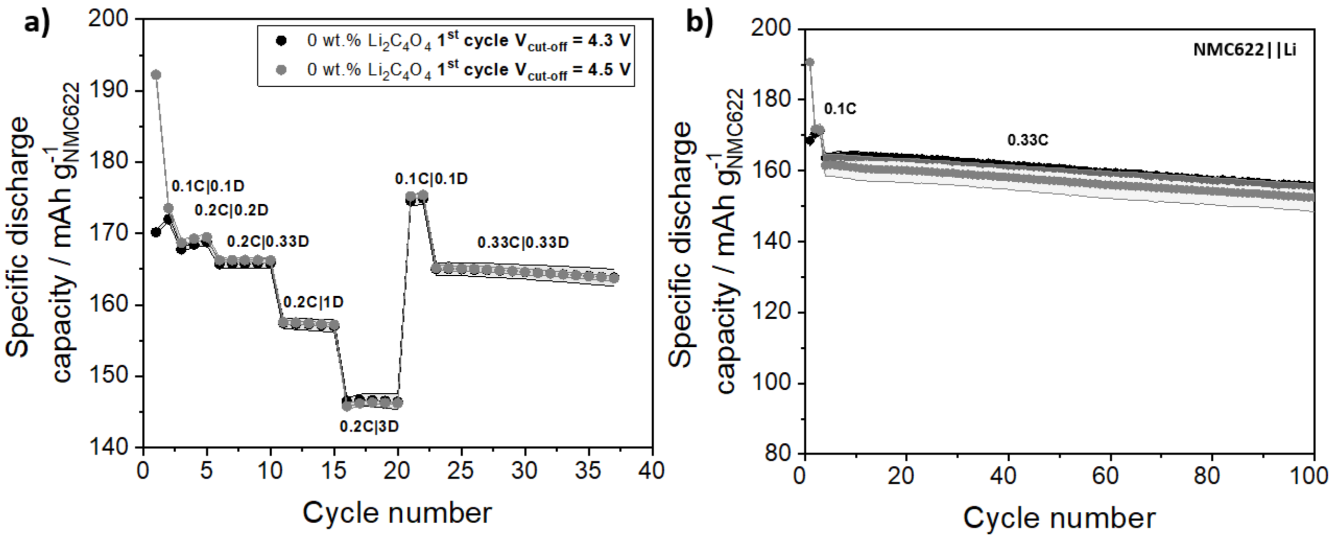

Figure S5. Evaluation of cycling performance: (a) rate capability and (b) short-term cycling of NMC622||Li metal cells without additive from 2.9 to 4.3 V (first cycle upper cut-off voltage of 4.3 or 4.5 V) with an active mass loading of ≈5 mg_{NMC622} cm^{-2}. For rate capability investigations, the C-rate was only varied upon discharge and kept at 0.2C during charge. Error bars represent the standard deviation of three cells. 1C = 170 mA g^{-1}.

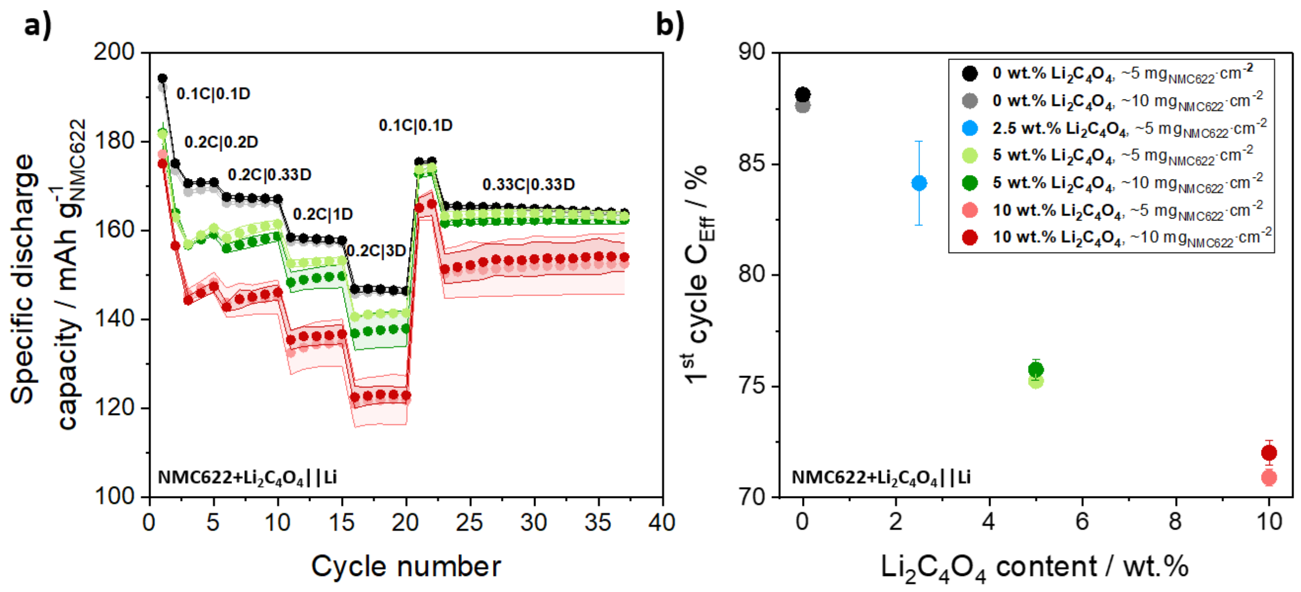

Figure S6. (a) Rate capability of NMC622+$Li_2C_4O_4$||Li metal cells with or without additive at different C-rates from 0.1C to 3C in a cell voltage window between 2.9 and 4.3 V (first cycle upper cut-off voltage of 4.5 V). The C-rate was only varied upon discharge and kept at 0.2C during charge. (b) First cycle Coulombic efficiencies (C_{Eff}) as a function of initial $Li_2C_4O_4$ content within the positive electrode. Active mass loadings (based on NMC622) of ≈5 and 10 mg_{NMC622} cm^{-2} are considered. Error bars represent the standard deviation of three cells tested for each sample.

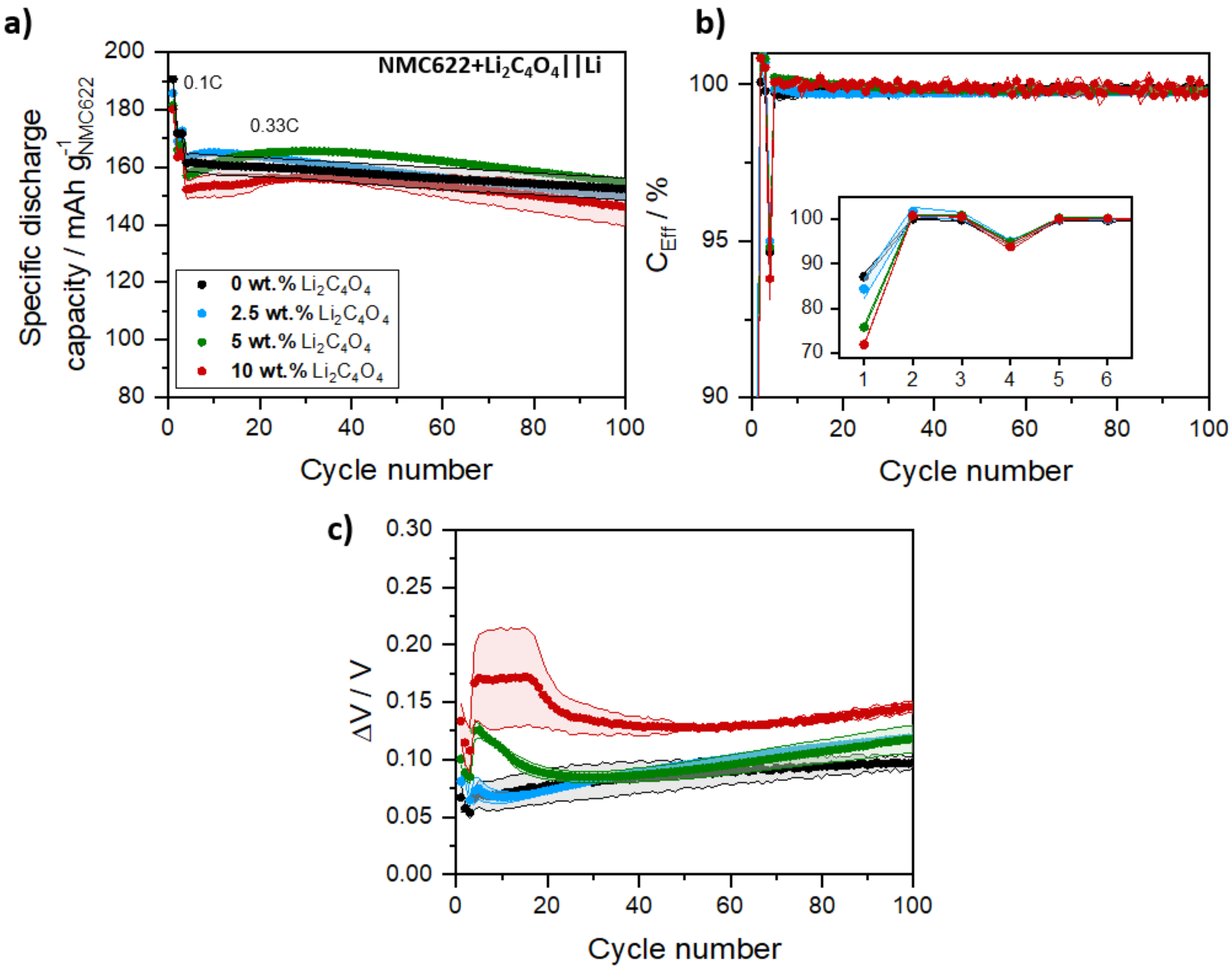

Figure S7. (a) Specific discharge capacities, (b) C_{Eff} and (c) difference between charge and discharge voltages (ΔV) of NMC622+$Li_2C_4O_4$||Li metal cells over cycling at 0.33C from 2.9 to 4.3 V (4.5 V in the first cycle) with an active mass loading (based on NMC622 weight) of ≈5 mg_{NMC622} cm^{-2}. Error bars represent the standard deviation of three cells tested for each sample.

Post-mortem XPS characterization of NMC622 electrodes from NMC622+$Li_2C_4O_4$||Li metal cells

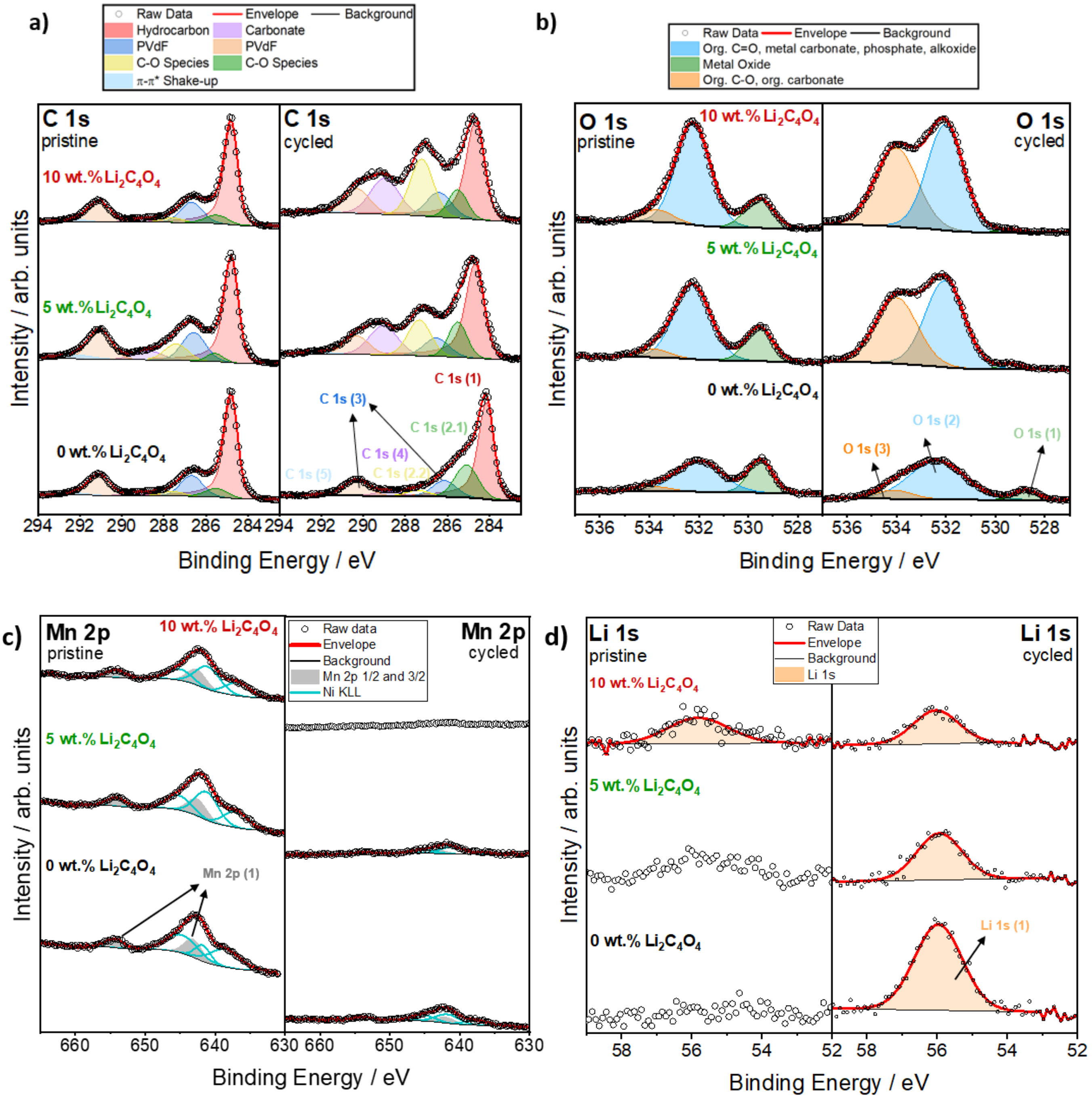

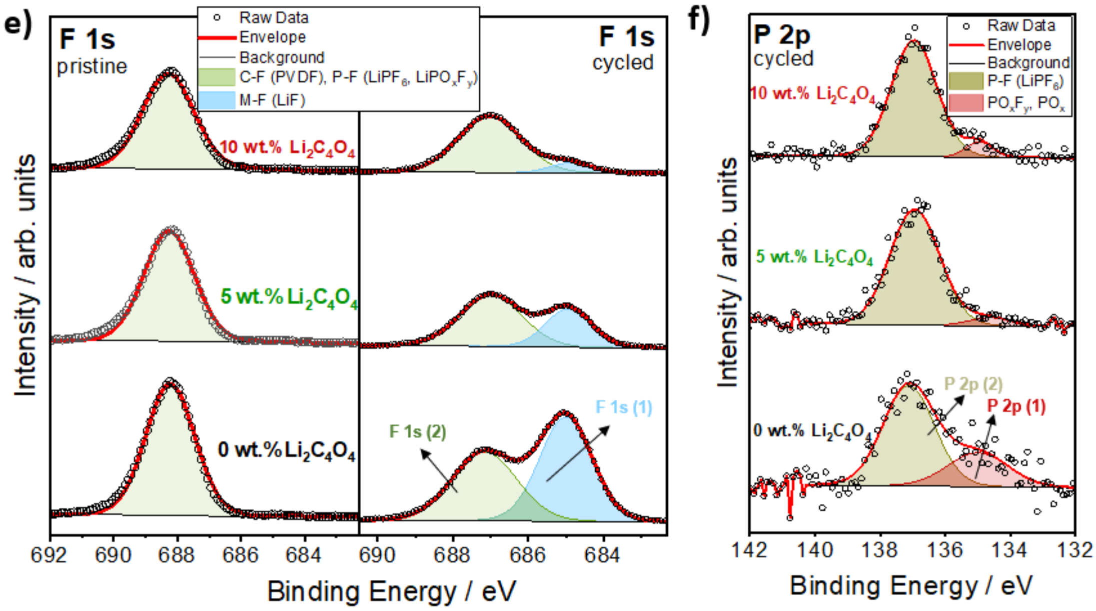

Figure S8. (a) C 1s, (b) O 1s, (c) Mn 2p, (d) Li 1s, (e) F 1s and (f) P 2p core spectra of pristine and cycled NMC622 positive electrodes comprising 0 wt.%, 5 wt.% and 10 wt.% $Li_2C_4O_4$. First cycle cell voltage range: 2.9-4.5 V in NMC622+$Li_2C_4O_4$||Li metal cells.

Electrochemical characterization of NMC622+$Li_2C_4O_4$||Si/graphite LIB cells

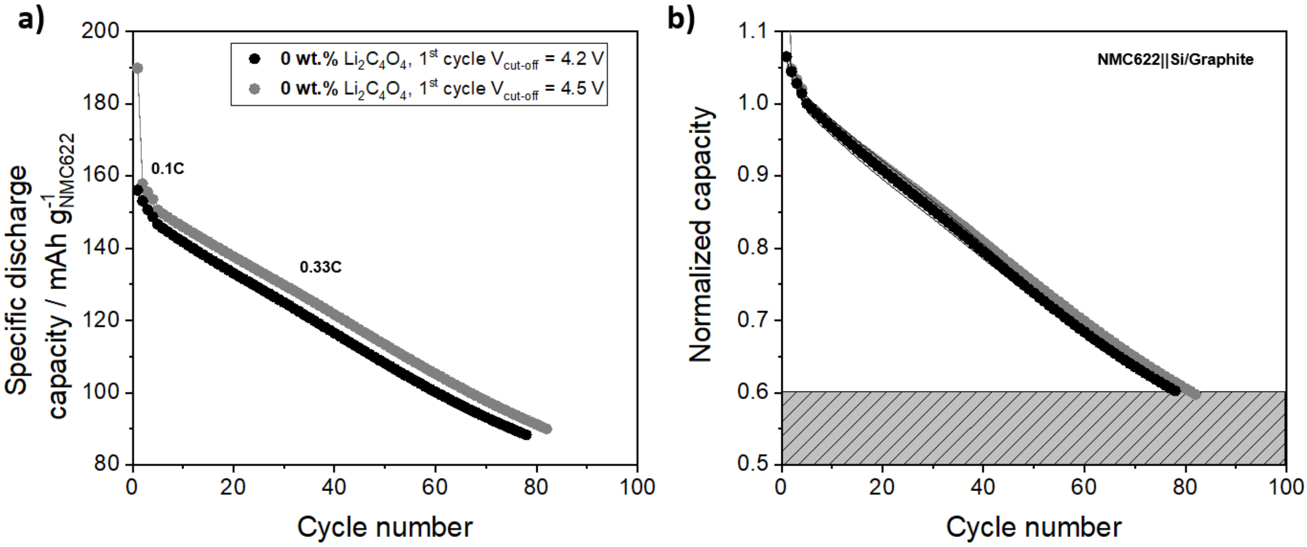

Figure S9. (a) Specific discharge capacity and (b) normalized capacity (calculated based on the 5th cycle discharge capacity) of the long-term cycling stability experiments in NMC622||Si/graphite LIB cells without pre-lithiation additive from 2.8 to 4.2 V (or 4.5 V for the first cycle) at 0.33C (1C = 170 mA g^{-1}). A composite of silicon nanowires and graphite served as negative electrode. Error bars represent the standard deviation of two cells tested for each sample. Cells stopped after reaching 60% SOH.

N/P capacity balancing:

In an ideal case with no parasitic reactions, electrode balancing should be stoichiometric (N/P ~ 1).[5] However, a practical graphite-based LIB full cell normally requires capacity-overbalancing of the negative electrode of up to 10-15% to ensure high energy density while preventing safety issues due to Li metal plating (*e.g.*, at low operating temperature or high charging rate).[6] Therefore, cells were overbalanced by a N:P ratio of 1.20:1.00, with the only exception of the cells containing 10 wt.% $Li_2C_4O_4$ that were overbalanced to 1.25:1.00. An overview of the resulting end-of-charge (EOC) and end-of-discharge potentials (EOD) for the positive and negative electrodes is given in **Table S1**. By considering a N/P ratio of 1.20:1.00 (**Figure S10**), cells containing 10 wt.% $Li_2C_4O_4$ reach anode potentials (*vs.* Li|Li^+) below 0 V in the first charge, giving evidence of Li metal plating. Due to active lithium consumption because of SEI formation at the anode surface and other occurring parasitic side reactions, the first cycle upper cathode potential of the electrode without additive (0 wt.% $Li_2C_4O_4$) is ≈0.06 V higher than the cell voltage, further indicating that the cathode is forced to operate at higher EOC potentials to promote a higher Li extraction ratio to compensate for active lithium losses. However, the difference between first cycle upper cathode potential and cell voltage becomes smaller in the additive containing cells (5 wt.% and 10 wt.% $Li_2C_4O_4$), since part of the lithium released by the pre-lithiation additive contributes to SEI formation at the anode surface. While a Li

reservoir is present within the cell, the anode is not completely emptied during discharge and remains at lower potentials. At the EOD cell voltage of 2.8 V, cathode potentials of the pre-lithiated cells drop rapidly caused by the polarization of the electrode, indicating that the amount of Li is completely restored due to the additional lithium provided by the pre-lithiation additive.[7]

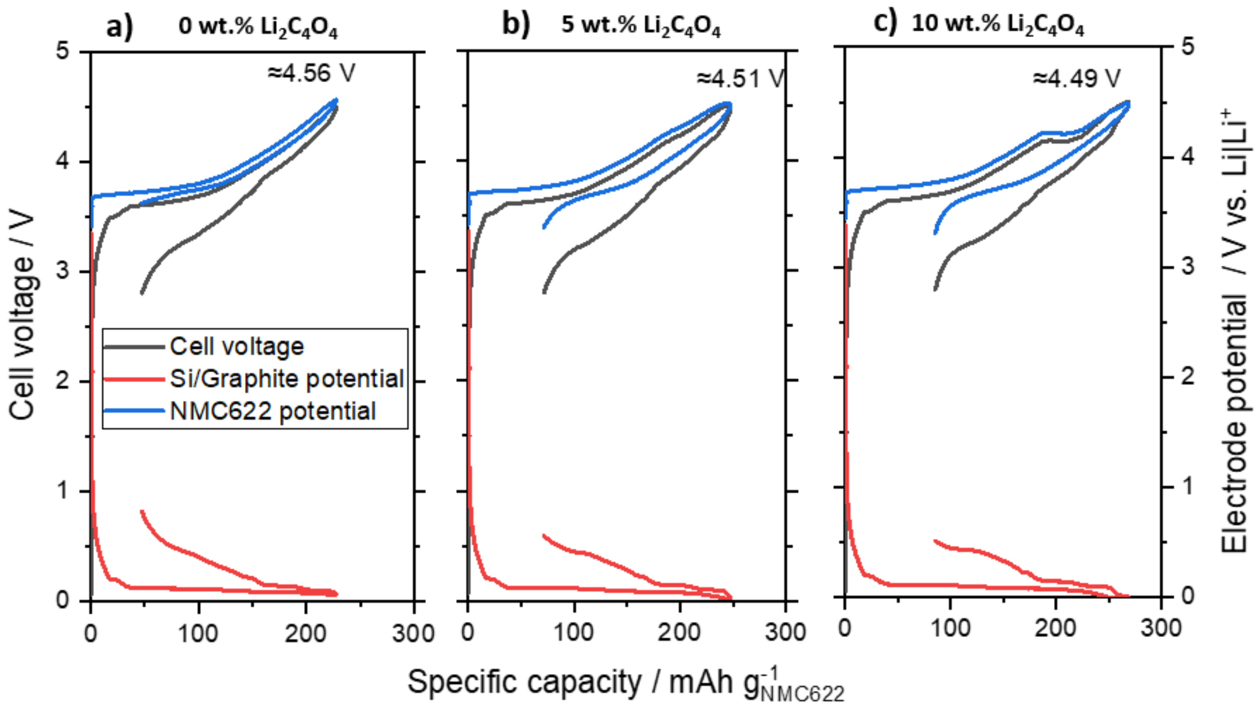

Figure S10. First cycle cell voltage and individual potential profiles of each electrode in NMC622+$Li_2C_4O_4$||Si/graphite full-cells at cut-off voltages of 2.8-4.5 V, with a CV step until the specific current reached values below 0.05C (1C = 170 mA g^{-1}): (a) 0 wt.% $Li_2C_4O_4$, (b) 5 wt.% $Li_2C_4O_4$ and (c) 10 wt.% $Li_2C_4O_4$ within the cathode. N:P ratio: 1.20:1.00.

Table S1. First cycle end-of-charge (EOC) and end-of-discharge (EOD) cell voltage and electrode potentials (in V *vs.* Li|Li^+) of the positive (NMC622+$Li_2C_4O_4$) and negative electrode (Si/graphite) in NMC622+$Li_2C_4O_4$||Si/graphite LIB cells within the cell voltage range 2.8-4.5 V. N:P balancing ratio of 1.20:1.00 or 1.25:1.00 (for 10 wt.% $Li_2C_4O_4$).

Sample	N:P balancing ratio	EOC cell / V	EOC anode / V *vs.* Li|Li^+	EOC cathode / V *vs.* Li|Li^+	EOD cell / V	EOD anode / V *vs.* Li|Li^+	EOD cathode / V *vs.* Li|Li^+
**0 wt.% $Li_2C_4O_4$**	1.20:1.00	4.5	0.06	4.56	2.8	0.82	3.62
**5 wt.% $Li_2C_4O_4$**	1.20:1.00	4.5	0.01	4.52	2.8	0.59	3.39
**10 wt.% $Li_2C_4O_4$**	1.20:1.00	4.5	-0.01	4.49	2.8	0.51	3.31
**10 wt.% $Li_2C_4O_4$**	1.25:1.00	4.5	0.01	4.51	2.8	0.45	3.25

__**EIS analysis:**__

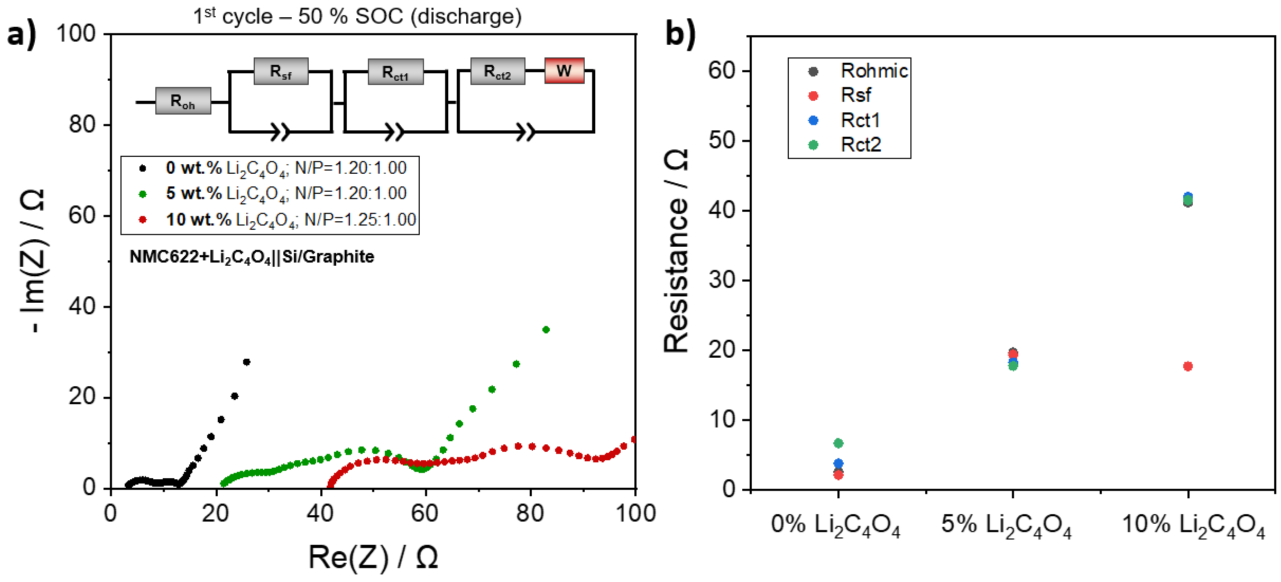

Figure S11. (a) Nyquist plots of the EIS measurements of the NMC622+$Li_2C_4O_4$||Si/graphite full-cells at 50 % SOC after the 1st cycle at 0.1C (1C = 170 mA g^{-1}). Cell voltage: 2.8–4.5 V. Equivalent circuit model for EIS data fitting is shown in the inlet of the figure. (b) Evolution of the resistances as determined by EIS fitting as a function of pre-lithiation additive content.

Electrochemical impedance spectroscopy (EIS) measurements were performed to investigate the impact of the pre-lithiation additive on the resistance of the cells after irreversible oxidation in the first cycle. Nyquist plots after the 1st cycle of NMC622+$Li_2C_4O_4$||Si/graphite full-cells at 50% state-of-charge (SOC) are depicted in **Figure S11a**. The equivalent circuit model used to fit the EIS data is also shown in the inlet of **Figure S11a**. The high frequency region of the spectra represents the Ohmic resistance of the cells (R_{oh}). The mid-frequency region of the Nyquist plots comprises the resistance of Li^+ migration through the interphases at the anode and cathode, double layer penetration and charge transfer resistance at the electrodes. In this study, surface film resistance (R_{sf}, or SEI resistance), charge transfer resistance I (Rct_1, or anode charge transfer resistance), and charge transfer resistance II (Rct_2, or cathode charge transfer resistance) were calculated, in agreement with An et al.[8] The low frequency range appears ideally as a straight line with a constant slope, which corresponds to the Warburg resistance (W) of solid state diffusion of Li^+ ions in the electrodes.[9] As can be seen in **Figure S11b**, the charge transfer and surface film resistances of the cells monotonically increase with the initial amount of pre-lithiation additive. These results are in agreement with the trend of the ΔV (**Figure 4f**), and can be further evidence that part of the released CO_2 gases can contribute to form a thicker CEI and increase the overall interfacial resistances of the cell.

Post mortem XPS characterization of NMC622 cathodes from NMC622+$Li_2C_4O_4$||Si/graphite cells

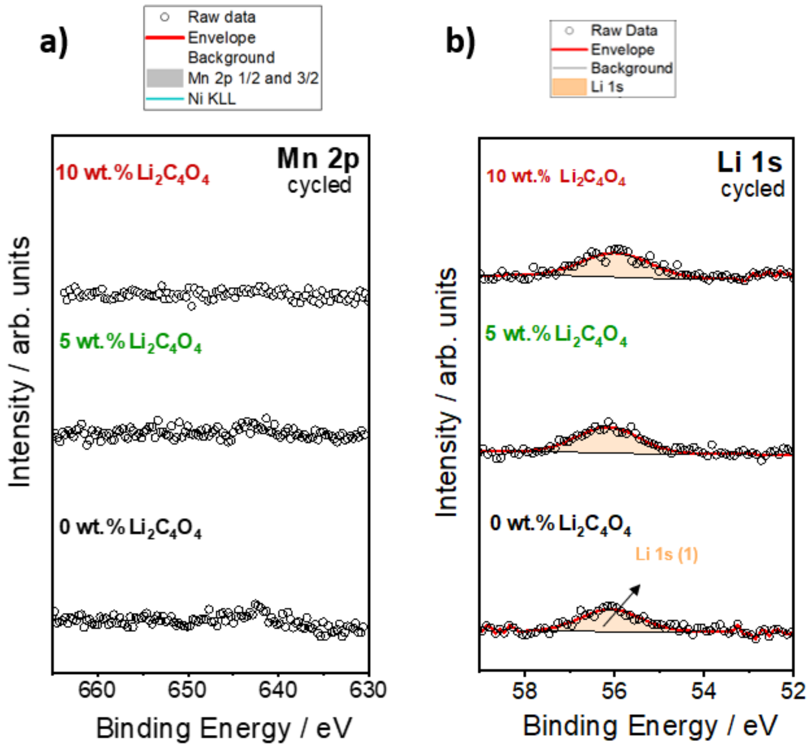

Figure S12. (a) Mn 2p and (d) Li 1s core spectra of cycled NMC622 positive electrodes comprising 0 wt.%, 5 wt.% and 10 wt.% $Li_2C_4O_4$. First cycle voltage range: 2.8-4.5 V in NMC622+$Li_2C_4O_4$||Si/graphite cells.

Post mortem XPS characterization of Si/graphite anodes from NMC622+$Li_2C_4O_4$||Si/graphite cells

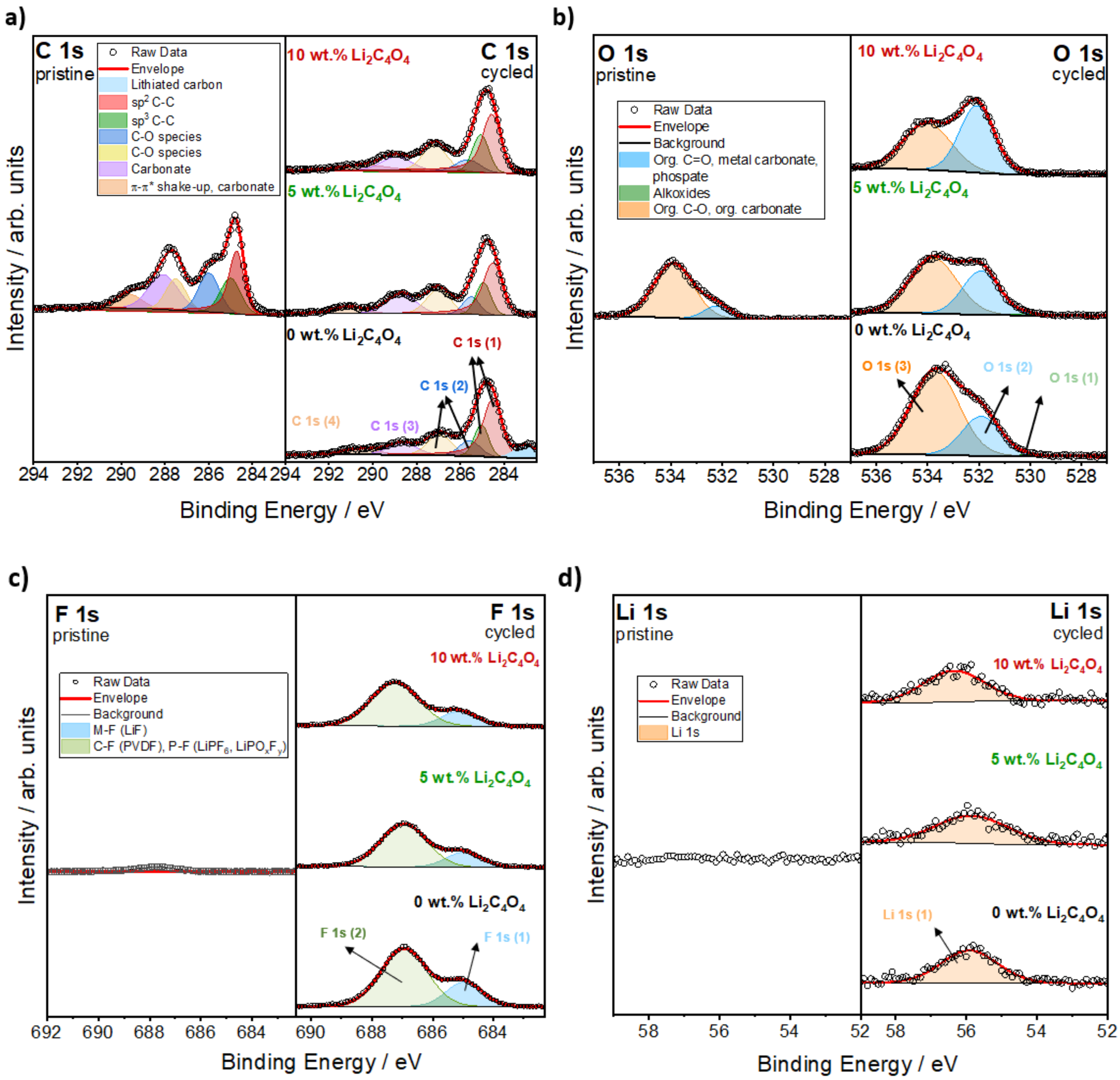

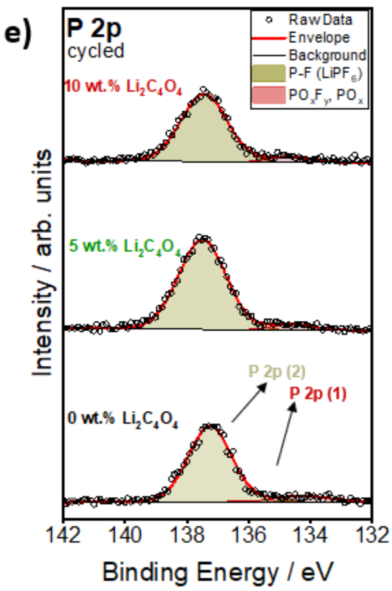

Figure S13. (a) C 1s, (b) O 1s, (c) F 1s, (d) Li 1s and (e) P 2p core spectra of pristine and cycled Si/graphite negative electrodes using 0 wt.%, 5 wt.% and 10 wt.% $Li_2C_4O_4$ as cathode additive. First cycle voltage range: 2.8-4.5 V in NMC622+$Li_2C_4O_4$||Si/graphite cells.

References

[1] J.-H. Kim, K.-J. Park, S. J. Kim, C. S. Yoon, Y.-K. Sun, *Journal of Materials Chemistry A* **2019**, 7, 2694.

[2] M. Arnaiz, D. Shanmukaraj, D. Carriazo, D. Bhattacharjya, A. Villaverde, M. Armand, J. Ajuria, *Energy & Environmental Science* **2020**, 13, 2441.

[3] a) J. Martínez De Ilarduya, L. Otaegui, M. Galcerán, L. Acebo, D. Shanmukaraj, T. Rojo, M. Armand, *Electrochimica Acta* **2019**, 321, 134693; b) Y.-B. Niu, Y.-J. Guo, Y.-X. Yin, S.-Y. Zhang, T. Wang, P. Wang, S. Xin, Y.-G. Guo, *Advanced Materials* **2020**, 32, 2001419.

[4] J. Syzdek, M. Marcinek, R. Kostecki, *Journal of Power Sources* **2014**, 245, 739.

[5] J. Kasnatscheew, T. Placke, B. Streipert, S. Rothermel, R. Wagner, P. Meister, I. C. Laskovic, M. Winter, *Journal of The Electrochemical Society* **2017**, 164, A2479.

[6] D. Andre, H. Hain, P. Lamp, F. Maglia, B. Stiaszny, *Journal of Materials Chemistry A* **2017**, 5, 17174.

[7] a) J. Kasnatscheew, U. Rodehorst, B. Streipert, S. Wiemers-Meyer, R. Jakelski, R. Wagner, I. C. Laskovic, M. Winter, *Journal of The Electrochemical Society* **2016**, 163, A2943; b) I. Buchberger, S. Seidlmayer, A. Pokharel, M. Piana, J. Hattendorff, P. Kudejova, R. Gilles, H. A. Gasteiger, *Journal of The Electrochemical Society* **2015**, 162, A2737.

[8] S. J. An, J. Li, C. Daniel, H. M. Meyer, S. E. Trask, B. J. Polzin, D. L. Wood, *ACS Applied Materials & Interfaces* **2017**, 9, 18799.

[9] D. Becker, M. Börner, A. Friesen, S. Klein, U. Rodehorst, M. Diehl, M. Winter, T. Placke, R. Schmuch, *Journal of The Electrochemical Society* **2020**, 167, 060524.
